# Does Changing Vertical Disparity Induce Horizontal Head Movement?

**DOI:** 10.1371/journal.pone.0137483

**Published:** 2015-09-10

**Authors:** Toru Maekawa, Hirohiko Kaneko

**Affiliations:** 1 Center for Information and Neural Networks, National Institute of Information and Communication Technology, Suita, Osaka, Japan; 2 Department of Information Processing, Interdisciplinary Graduate School of Science and Engineering, Tokyo Institute of Technology, Yokohama, Kanagawa, Japan; University of Münster, GERMANY

## Abstract

Theoretically, one can estimate the direction of an object that is relative to the head using vertical disparity if the distance from the head to the object is known. However, several reports describe vertical disparity as having little or no effect on the perception of visual direction. It has been suggested, however, that the visual processes involved in action are different from those involved in perception, and the effect of visual disparity on action has not been investigated in previous studies. This study investigated the influence of vertical disparity on the stability of head direction as a motor response to visual information. We presented a stimulus consisting of horizontal lines with vertical size-disparity oscillation, and examined whether the stimulus affected the subject’s head movement. The results showed that the head movement in the condition of vertical size-disparity oscillation was not significantly different from that in the condition of no disparity oscillation. Our results suggest that, despite theoretical validity, vertical disparity is not used for controlling head movement.

## Introduction

Humans can estimate the direction of objects relative to the head or body in the visual field, which is necessary for perceiving the position of objects relative to them and for performing actions such as grasping or rotating the head toward the object. When humans estimate the direction of objects relative to the head in the visual field, the visual system has to know the direction of the eyes relative to the head. There are two main cues to estimating the direction of the eyes. One is extra-retinal eye position signals, and the other is retinal signals including binocular disparity and visual references, such as the nose and eyelids. Most previous research has focused on eye position signals as the main or only cue for estimating eye direction [1–4], and the role of retinal signals has not yet been thoroughly investigated.

Vertical disparity refers to the vertical component of binocular disparity. It has been suggested that the human visual system uses vertical disparity to estimate the direction of an object that is relative to the head when the distance to the object is known or when the relative positions of the objects are known [5–8]. This is understandable, intuitively, if one considers the difference in the distances from both eyes to an object at an eccentric location. If an object is placed on the left side of the head’s median plane, the retinal image in the left eye becomes larger than that in the right eye because the distance from the left eye is less than that from the right eye. This overall size disparity, the difference in the right and left eye images, is divisible into horizontal and vertical components.

**Fig 1:**
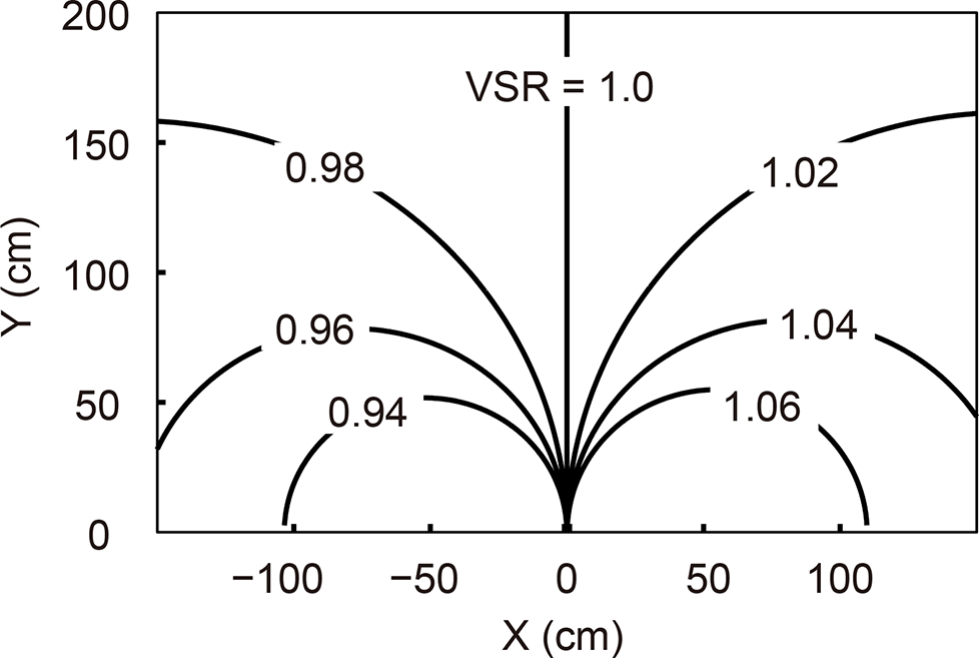
Overhead view through the visual plane. Each line is a contour that shows the vertical size ratio to be constant. Each is labeled with its VSR (V_R_/V_L_).

The vertical size ratio (VSR), which is the ratio of vertical angles at the two eyes, is a convenient measure of vertical disparity under such circumstances [9,10]. Conventionally, the VSR is defined as the ratio of the right eye’s vertical image size (V_R_) to the left eye’s vertical image size (V_L_). Fig 1 demonstrates the loci in space for which the VSR is constant. As shown in the figure, the VSR (V_R_ / V_L_) is related to both direction and distance. Since the distance to an object can be estimated from vergence or from other cues [5,8], the visual direction of an object can be estimated using VSR information.

Experimentally, however, previous reports have suggested that the VSR has little or no effect on the perception of visual direction. Banks et al. [6] asked subjects to respond to the direction of a visual target using a manual pointer while manipulating the VSR of the target. They report that the VSR does not affect the perceived direction of the target. Berends et al. [7] asked subjects to judge the target position as *left* or *right*, relative to the median plane, using a stimulus plane consisting of random dots with several VSR conditions. Their results also show that the VSR does not affect the perceived direction, although adaptation to the VSR over a long time (5–10 minutes) has a slight effect.

However, these previous studies only account for visual perception and did not investigate any effect on action. The visual control of action has been proposed as distinct from visual perception [11]. In the field of binocular disparity processing, Uwa et al. [12,13] presented experimental results that are consistent with this view. They presented to subjects a stimulus simulating a front parallel wall oscillating backward and forward. Then, they measured the perceived movement of the wall and body sway. The movement of the wall was defined by pictorial depth cues and/or binocular disparity. Results of that study revealed that the movement defined by pictorial cues produces a strong perception of surface movement and little body sway. However, the movement defined by the binocular disparity produced a weak perception of surface movement and a large body sway. Erkelens and Collewijn [14] also reported that changing disparity of a large frontal surface produced weak perception of surface movement but induced vergence eye movement that is almost as great as the value predicted from the geometry. Additionally, we found static vertical disparity to have a minor effect on the goal of the head pointing to an object [15]. Based on these previous results, we considered the possibility that the VSR can affect action even if there is little effect on perception.

We have to stabilize the body in the space on the earth. Because the eyes, head, and body can move separately, so to speak, maintaining stability in each coordinate is reasonable. Certainly, reflex to a visual stimulus occurs for eye and body movements. In the present study, we assumed that the head also responds to visual stimulus for maintaining stability; however, as far as we know, reflex movement of the head produced by a visual stimulus has not been reported. Therefore, we investigated whether the VSR can affect head movement about the vertical axis.

**Fig 2:**
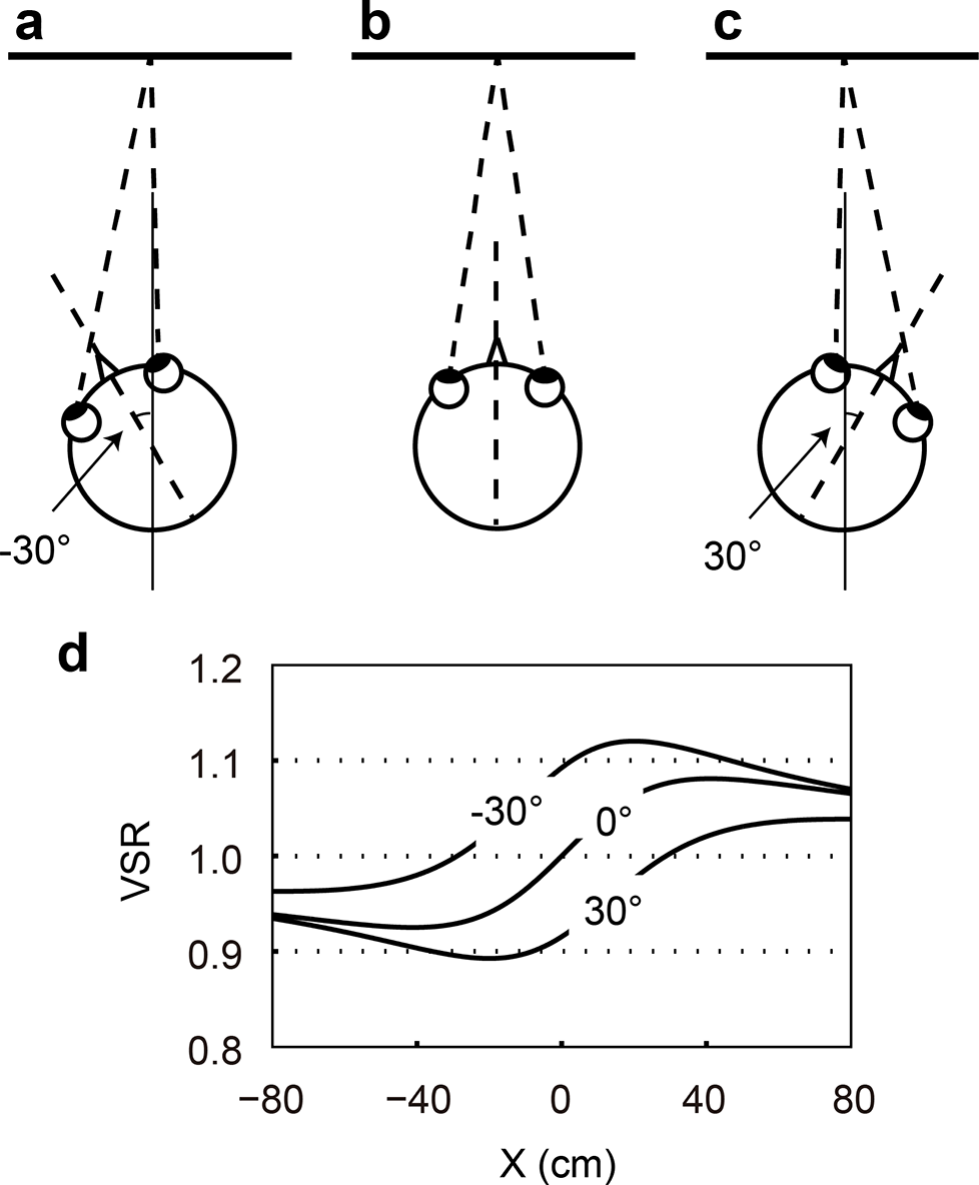
Schematic top view of subject and a front parallel plane for different conditions of head direction: (a) −30°, (b) 0°, and (c) 30° (d) VSR produced by the front parallel plane for each head direction condition. The horizontal axis shows the horizontal position on the frontal parallel plane. The viewing distance was set at 50 cm for the calculation.

Fig 2A, 2B and 2C, respectively, show schematic diagrams of situations with different horizontal head directions in front of a stationary frontal plane while fixating at a point on the surface. Fig 2D demonstrates the distribution of vertical disparity near the horizontal meridian in each of the head direction conditions. In the case of a −30° head direction (rotated to the left), the VSR is larger than that in the case of a 0° head direction at any point on the surface. In the case of a 30° head direction (rotated to the right), the VSR is less than that in the case of a 0° head direction at any point on the surface. In short, the VSR increases or decreases throughout the visual field according to the direction and magnitude of head rotation. This fact means that the sign and magnitude of the VSR can indicate the head direction, and that the temporal change of the VSR can be a signal for self-monitoring head rotation. Assuming that such a mechanism does exist, the visual system does not need access to accurate information about the visual distance in order to judge the direction of head rotation, and so the visual system would be able to respond quickly. Thus, this mechanism of the visual system could potentially control head rotation, at least partly.

This study investigated the influence of changing vertical disparity on the control of head direction. As described above, vertical disparity ratio (VSR) can indicate head direction theoretically. We investigated whether a task that kept the head directing straight toward an object with changing VSR could induce head movement. We conjectured that vertical disparity might affect head movement, even if it did not affect the judgment of direction as reported in earlier studies, because of differences in visual processing for perception and action. We presented to subjects a stimulus consisting of horizontal lines with vertical disparity oscillations, and we measured the head rotation about the vertical axis while subjects viewed the stimulus. Our results showed that the temporal change of the VSR did not affect the subject’s head direction in any of the experiments. The results suggest that, as in the case of visual perception, changes in vertical disparity do not affect head movement control.

## Methods

**Fig 3:**
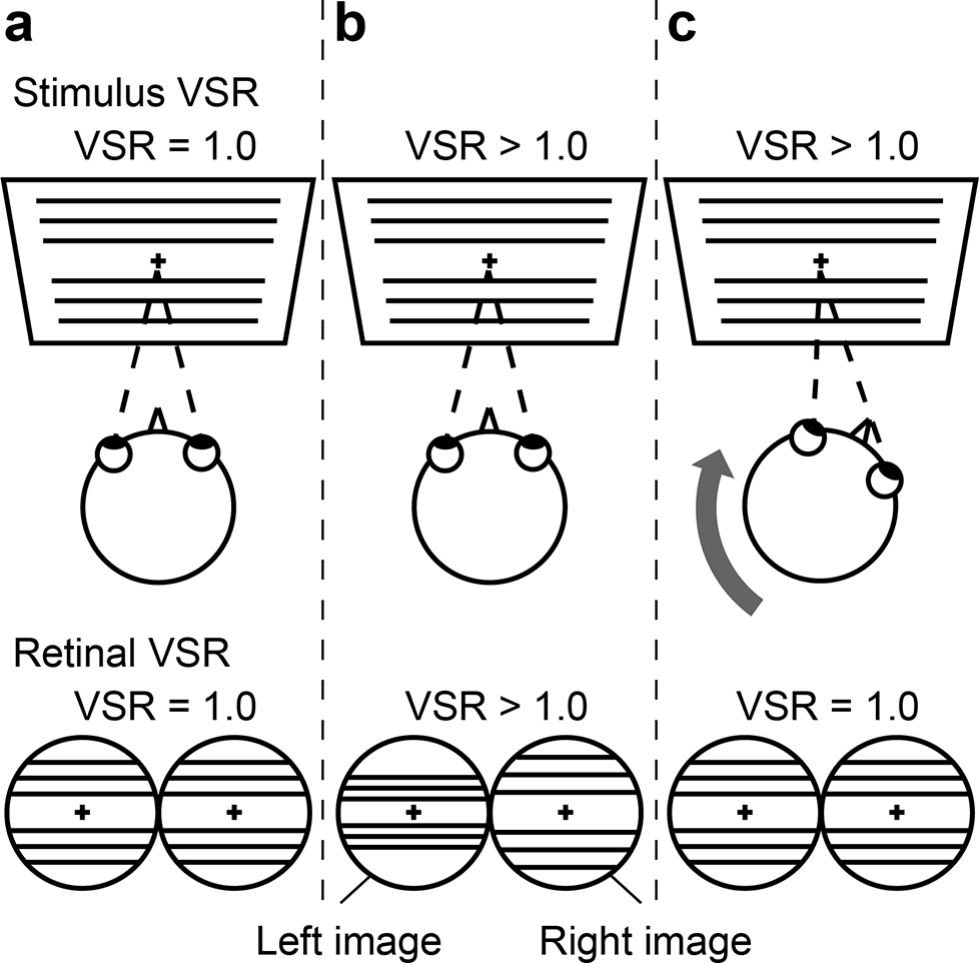
Schematic diagram of head movement induced by a change of VSR. The upper image shows a top view of the subject’s head and screen. The lower two circles represent images of the subject’s retinas.

This experiment was conducted to investigate whether temporal changes in the VSR induced head movement. Fig 3 demonstrates the experimental setup, and an example of the head movement needed for the subject to maintain constant retinal VSR in response to changes in the stimulus VSR. In this experiment, the subject faced and looked at a fixation point on the median plane of the head. The stimulus VSR was 1.0 around the fixation point (Fig 3A). When the stimulus VSR was slightly increased (Fig 3B), the retinal disparity was the same as if the fixation point had moved to the right side of the median plane, or if the head had turned slightly to the left. If retinal VSR is used for controlling the head position toward the fixation point, then the subject needed to move its head toward the right in order to maintain a constant VSR at 1.0 (Fig 3C).

### Apparatus

Each subject sat behind a large screen (160 cm × 120 cm, 116° × 100°, 1024 × 768 pixels) on which the stimulus was presented. Two projectors (LP-W1000; Sanyo Electric Co. Ltd.) were used to present the images to both eyes. A pair of polarizing filters in front of the projectors and glasses with polarizing filters were used to present the images dichoptically. The subjects wore a helmet on which four LED targets were mounted. We measured the movement of the LED targets on the plane that was parallel to the ground using a position sensor (C5949; Hamamatsu Photonics KK) placed 50 cm above the subject’s head. The LED positions were recorded with a temporal frequency of 10 Hz. A horizontal pole attached to two pillars was put onto the subject’s chest, and the subject was instructed to continue touching the pole during the experiments to prevent body movement. The distance from the pole to the screen was 50 cm. The height of the bar from the floor was adjusted for each subject.

### Stimuli

The stimulus image consisted of 20 horizontal lines and a fixation point, as schematized in Fig 3. The luminance gradient along the width of the line had the shape of a Gaussian distribution with a standard deviation of 2.0 pixels, which was 0.36° of visual angle at eye height. The fixation point was presented at eye level. This horizontal line image was used to prevent the subject from using horizontal disparity in order to enable the investigation of the influence of vertical disparity alone.

The stimulus VSR was defined as the vertical height of the right eye’s image (V_R_) relative to that of the left eye’s image (V_L_) (VSR = V_R_ / V_L_). In the conditions where the stimulus VSR was changed, the vertical height of one image was magnified VSR^1/2^ times and that of the other image was reduced VSR^-1/2^ times relative to that of the original image (V_0_), (V_R_ = *V*_0_ × VSR^1/2^, V_L_ = *V*_0_ / VSR^1/2^ then, V_R_ / V_L_ = (VSR^1/2^)^2^ = VSR). The position of each line was changed according to the VSR value. The line position was manipulated with a 1/100 sub-pixel resolution using an anti-aliasing technique.

During each trial, the VSR oscillated sinusoidally. The temporal oscillation was defined by the following equation:

$$VSR(t)=1+({VSR}_{\text{max}}-1)\;\text{sin}(2\pi \frac{t}{T}+\alpha )$$ (1)

In this equation, *T* denotes the period of one cycle, *α* stands for the initial phase, and *VSR*_*max*_ signifies the amplitude of the disparity oscillation.

In each trial, the fixation point was presented on the screen first. Then, the subject turned its head to the fixation point and pushed a button to initiate the stimulus presentation. Horizontal lines then appeared on the screen and oscillated sinusoidally according to Eq 1. The position of the subject’s head was recorded while the VSR oscillated. The amplitude of the VSR oscillation (*VSR*_*max*_) was 1.02, which is consistent with the head rotating about 8.9° while maintaining fixation on a stationary point in front of the nose. The initial phase (*α*) was 90°. The period (*T*) was 4 s or 8 s. Each trial lasted 140 s. We repeated three trials for each condition with each subject.

### Subjects

Twelve male adults, including one of the authors and eleven students selected from Tokyo Institute of Technology, volunteered to participate in this study. They were divided into two groups. Subjects in one group (Group A) were instructed to keep directing their heads toward the fixation point. Another group of subjects (Group B) were instructed to keep looking directly at the fixation point. The second set of instructions was used as a control to allow for the possibility of head movements being exaggerated or suppressed due to subjects being overly self-aware of their head position. There were seven subjects in the first group, and five in the second group. All subjects had normal or corrected-to-normal vision.

The subjects provided written informed consent for all examinations and procedures. Because the research involved negligible risks and no nominative/identifying information was collected, ethics approval was not required, and no IRB was consulted before conducting the study. Besides information concerning eyesight and age, no health information was collected from subjects.

## Results

We calculated the subject’s horizontal head direction from the positions of the four LED targets. The base head direction (0°) was defined as the averaged angle of the head direction for each trial, and positive angles corresponded to clockwise rotation. We eliminated data of the initial 15 s to avoid the influence of the trial onset (by the subjects’ operation of the button to initiate a trial and/or by the starting of the VSR oscillation). Indeed, some subjects moved largely to the right after the onset of the trial. We also eliminated the concluding 5 s to make the duration to be a multiple of 8 s for conducting frequency analyses. To investigate whether the subject’s head moved periodically due to an oscillating VSR, fast Fourier transform (FFT) and autocorrelation analyses were conducted.

**Fig 4:**
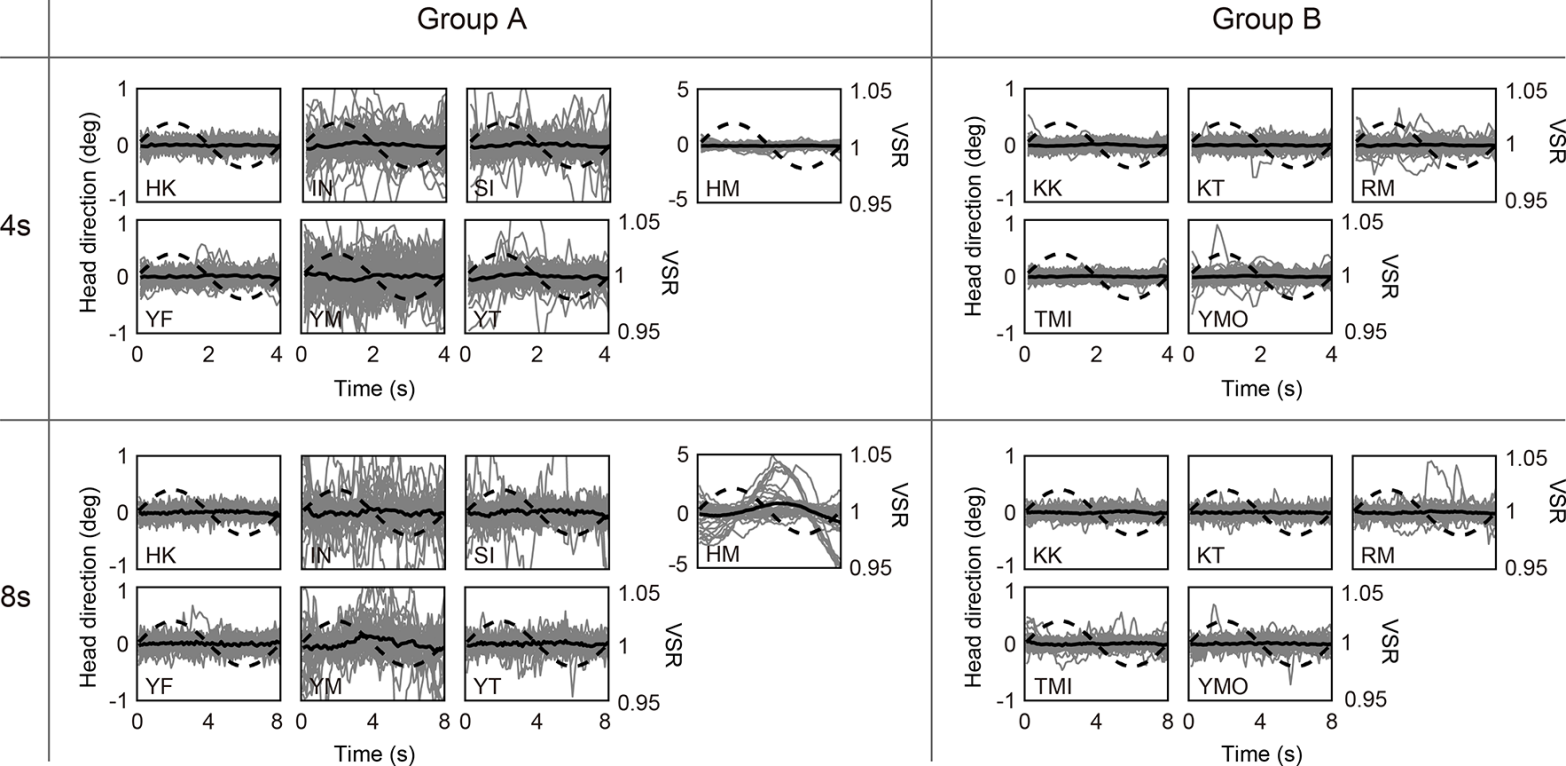
Divided head direction by one cycle of VSR oscillation. Gray lines show the head direction of one cut of the data. The thick black line shows the averaged head movement. The dotted line shows the presented VSR. The left-hand figures show the results under the instruction of “facing” and those on the right show those under the instruction of “looking.” The upper figures show the results of the 4-second condition for the oscillation period and the lower figures show those for 8-second condition.

First, the original raw measurements of the subject’s head angle were observed to check whether any visually clear head movement took place. Fig 4 shows the subjects’ head angles divided by the VSR oscillation period. Gray lines show one cut of the data, that is, there are 120 (s) / 4 (s) * 3 (trials) = 90 gray lines in one figure of the 4-second condition, and 120 (s) / 8 (s) * 3 (trials) = 45 gray lines in one figure of the 8-second condition. Black lines show the averaged value of gray lines. Dotted lines show the stimulus VSR, and the right y-axis shows its range. Almost all of the subjects did not make consistent head movements relative to the period of VSR oscillations. However, only subject HM made clear head movements within the same period as the VSR oscillation in the 8-second condition. Note that the y-axes are larger in subject HM than in other subjects because of his large head movements. Thus, we did not find any influence of VSR oscillations in the raw data, with the exception of subject HM whom we will discuss later. In addition, no differences were observed between groups A and B.

**Fig 5:**
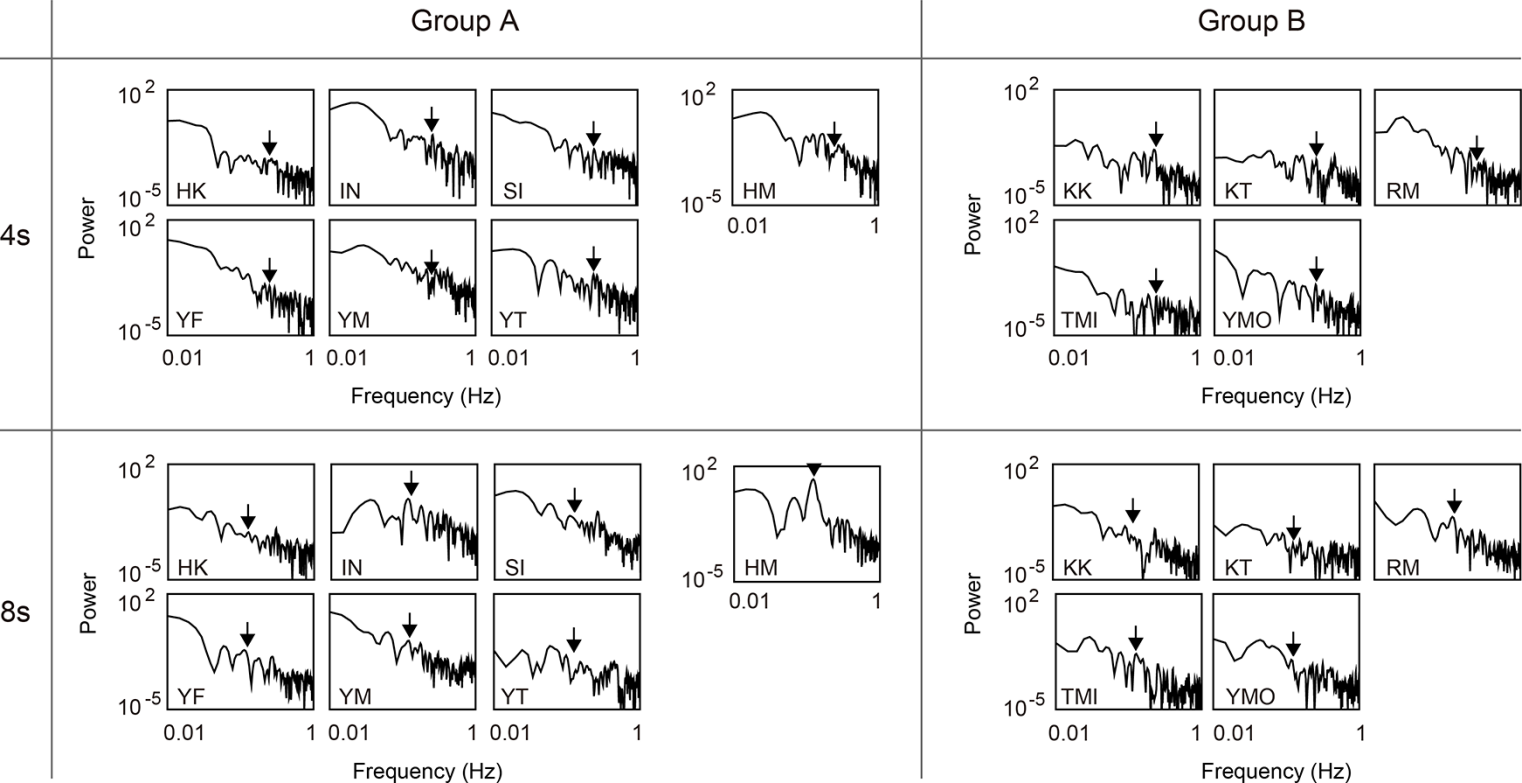
FFT power spectra of subjects’ head direction in Experiment 1. Each figure shows the averaged data for each subject. The left-hand figures show the results under the instruction of “facing” and those on the right show those under the instruction of “looking.” The upper figures show the results of the 4-second condition for the oscillation period and the lower figures show those for the 8-second condition. The arrows indicate the frequency of VSR oscillation.

Next, a Fourier analysis was conducted to investigate whether the subjects moved their heads at frequencies other than that of the VSR oscillation. We assume that Fourier analysis is the most basic and popular method for investigating the properties of oscillations contained in time sequential data. We computed the power spectrum of the subject’s angular head position using an FFT with a Blackman window. We calculated the FFT for each trial and averaged the power spectrum across all trials for each subject and condition. Fig 5 shows the resulting averaged power spectrum for each subject and condition (upper panels show results for the 4-second condition and lower panels show those of the 8-second condition). Black arrows indicate the VSR oscillation frequency. No clear peak was apparent at the VSR oscillation frequency (with the exception of HM), and no differences were observed between groups A and B. Consequently, periodical head rotation associated with VSR oscillation was not clearly observed from the FFT analysis.

**Fig 6:**
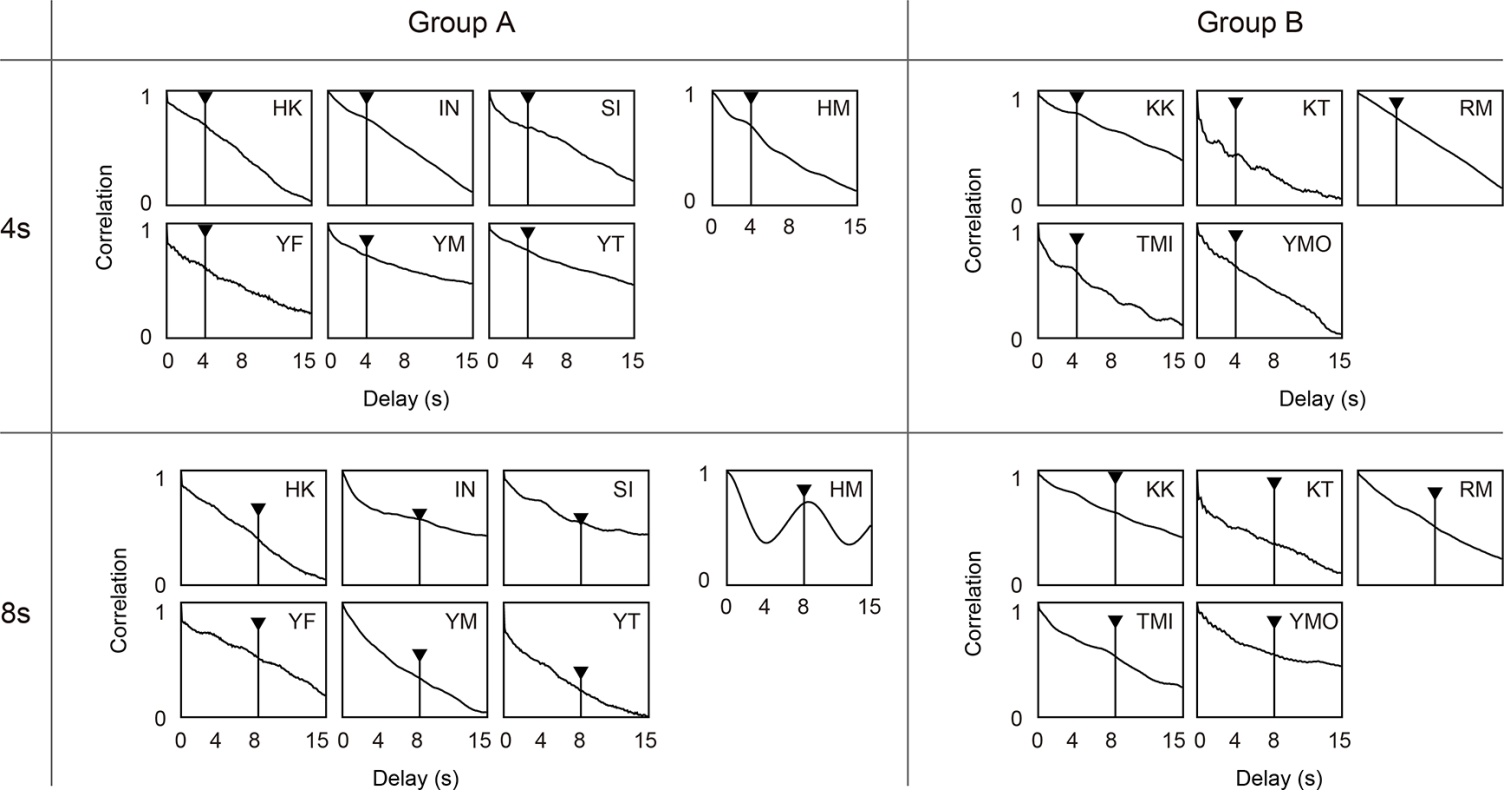
Autocorrelation of subjects’ head direction in Experiment 1. Each figure shows the averaged data for each subject. The left-hand figures show the results under the instruction of “facing” and those on the right show those under the instruction of “looking.” Upper figures show the results of the 4-second condition for the oscillation period, and the lower figures show those for the 8-second condition. The arrows indicate the period of VSR oscillation.

Finally, we applied an autocorrelation analysis to investigate the periodicity of the head movement. An autocorrelation analysis is suitable when the data has periodic structure but is not sinusoidal; for example, when the data has a single peak at a fixed time within each period. Using an autocorrelation analysis, we considered that effects overlooked by a FFT might be manifested. Fig 6 shows the result of the autocorrelation analysis. The *x*-axis shows the time lag and the black arrows indicate the VSR oscillation period. As in the case of the FFT analysis, clear peaks were not observed within the VSR oscillation period (with the exception of subject HM).

As described above, HM was the only subject whose data showed the VSR to have a clear effect on head movement. According to Fig 4, the phase of his sinusoidal head movement largely differed from the phase that was expected from VSR geometry. In addition, the amplitude of the head oscillation gradually increased to reach about 5°, which was over 10 times greater than those of other subjects. Some previous studies show that the phase of body sway induced by periodical optical flow stimulation approximately matches the stimulus’ phase when the frequency is lower than 0.2 Hz [16,17]. Subject HM stated that he had noticed his head movement, and that he had felt an impulse to rotate his head while viewing the stimulus. We conjecture that he rotated his head more or less voluntarily because he anticipated the purpose of the experiment from the stimulus and experimental setup. Therefore, we conclude that HM’s results were not due to the general effect of the VSR.

In summary, the result of this experiment suggests that vertical retinal disparity is not used to control head position in response to an oscillating VSR. This result is consistent with those of previous studies showing that the VSR did not affect the perception of visual direction [6,7], but differs from previous studies showing binocular disparity to have an effect on action [12,13,15].

## Discussion

We examined whether temporal oscillation of the vertical size ratio (VSR) induced the subject’s unconscious head movement. Geometrically, VSR can be a cue for maintaining the head direction relative to a stationary object and the temporal oscillation of VSR can produce head rotation about the vertical axis as explained in the introduction. The results, however, did not show VSR oscillation to have an effect on head movement.

Several previous studies report that binocular disparity affects body control, even though the disparity does not produce a perception of depth or direction [12,13,15]. In this study, we investigated whether changes in the VSR could induce passive head movement, even though the VSR does not produce a perception of the spatial position of an object. However, we found no significant effect of VSR on head movement. One possible explanation of this result could be that muscle proprioceptive information has a large effect. Ishii [18] reports that vertical disparity affects the perception of direction when proprioception cues from the eye muscles are weakened by an adaptation. This suggests that the visual system might use vertical disparity more when the other directional cues are not reliable. In the current experiments, the proprioceptive cues from eye and neck muscles would be relatively strong because the head position was almost stationary. Therefore, the sensory system may have relied primarily on proprioceptive information to estimate visual direction and ignored the changes in the VSR.

In principle, vertical disparity could be used for estimating direction. It has been shown that there is little or no effect of vertical disparity on the perception of stimulus direction, but the effect on vision for action has not been investigated until now. We investigated whether vertical disparity affects vision for action by measuring head movements while subjects viewed stimuli with an oscillating VSR. We did not, however, find clear and consistent effects of the VSR on head movement. The results suggest that vertical disparity has no discernible effect on direction estimation even for visual action.

## Supporting Information

S1 Fig Subjects' head movement in each trial. (PDF)

S1 Table Subjects' head movement in each trial. (XLSX)

## References

[1] Hering E. Spatial sense and movements of the eye Oxford: American Academy of Optometry; 1942.

[2] Nakamizo S, Shimono K, Kondo M, Ono H. Visual directions of two stimuli in Panum’s limiting case. Perception. 1994; 23: 1037–1048. 7899045 10.1068/p231037

[3] Ono H, Mapp AP. A restatement and modification of Wells-Hering’s laws of visual direction. Perception. 1995; 24: 237–252. 7617427 10.1068/p240237

[4] Wade N. An Essay Upon Single Vision with Two Eyes Destined for Distinguished Oblivion. Springer 2003; 71–117.

[5] Backus BT, Banks MS, van Ee R, Crowell JA. Horizontal and vertical disparity, eye position, and stereoscopic slant perception. Vision Res. 1999; 39: 1143–1170. 10343832 10.1016/s0042-6989(98)00139-4

[6] Banks MS, Backus BT, Banks RS. Is vertical disparity used to determine azimuth? Vision Res. 2002; 42: 801–807. 11927346 10.1016/s0042-6989(01)00283-8

[7] Berends EM, van Ee R, Erkelens CJ. Vertical disparity can alter perceived direction. Perception. 2002; 31: 1323–1333. 12489769 10.1068/p3440

[8] Mayhew JE, Longuet-Higgins HC. A computational model of binocular depth perception. Nature. 1982; 297: 376–378. 7078648 10.1038/297376a0

[9] Gillam B, Lawergren B. The induced effect, vertical disparity, and stereoscopic theory. Percept Psychophys. 1983; 34: 121–130. 6634367 10.3758/bf03211336

[10] Rogers BJ, Bradshaw MF. Vertical disparities, differential perspective and binocular stereopsis. Nature. 1993; 361: 253–255. 8423851 10.1038/361253a0

[11] Goodale MA, Milner AD. Separate visual pathways for perception and action. Trends Neurosci. 1992; 15: 20–25. 1374953 10.1016/0166-2236(92)90344-8

[12] Uwa N, Kaneko H, Kanatsugu Y. Human Visual and Auditory Information. Body Sway and Motion in Depth while Observing a Stimulus with Changing Disparity and Changing Size. The Journal of the Institute of Image Information and Television Engineers. 1999; 53: 1300–1307.

[13] Uwa N, Kaneko H. Body sway induced by changing-disparity and changing-size cues. Perception. 1998; 27 ECVP Abstract Supplement: 49. 10505203

[14] Erkelens CJ, Collewijn H. Motion perception during dichoptic viewing of moving random-dot stereograms. Vision Res. 1985; 25: 583–588. 4060612 10.1016/0042-6989(85)90164-6

[15] Maekawa T, Kaneko H. Effect of Binocular Disparity on Head Pointing. Vision. 2014; 26: 109–121.

[16] Lestienne F, Soechting J, Berthoz A. Postural readjustments induced by linear motion of visual scenes. Exp Brain Res.1977; 28: 363–384. 885185 10.1007/BF00235717

[17] Oie KS, Kiemel T, Jeka JJ. Multisensory fusion: simultaneous re-weighting of vision and touch for the control of human posture. Cognitive Brain Res. 2002; 14: 164–176. 10.1016/s0926-6410(02)00071-x 12063140

[18] Ishii M. Effect of a disparity pattern on the perception of direction: non-retinal information masks retinal information. Vision Res. 2009; 49: 1563–1568. doi: 10.1016/j.visres.2009.03.012 19324065 10.1016/j.visres.2009.03.012

