# Supplementary material for: Does Changing Vertical Disparity Induce Horizontal Head Movement?
Source: PLoS One. 2015 Sep 10;10(9):e0137483. doi: 10.1371/journal.pone.0137483 (PMC4565696; doi:10.1371/journal.pone.0137483)
Supplement: S1 Fig — (PDF) [file pone.0137483.s001.pdf]

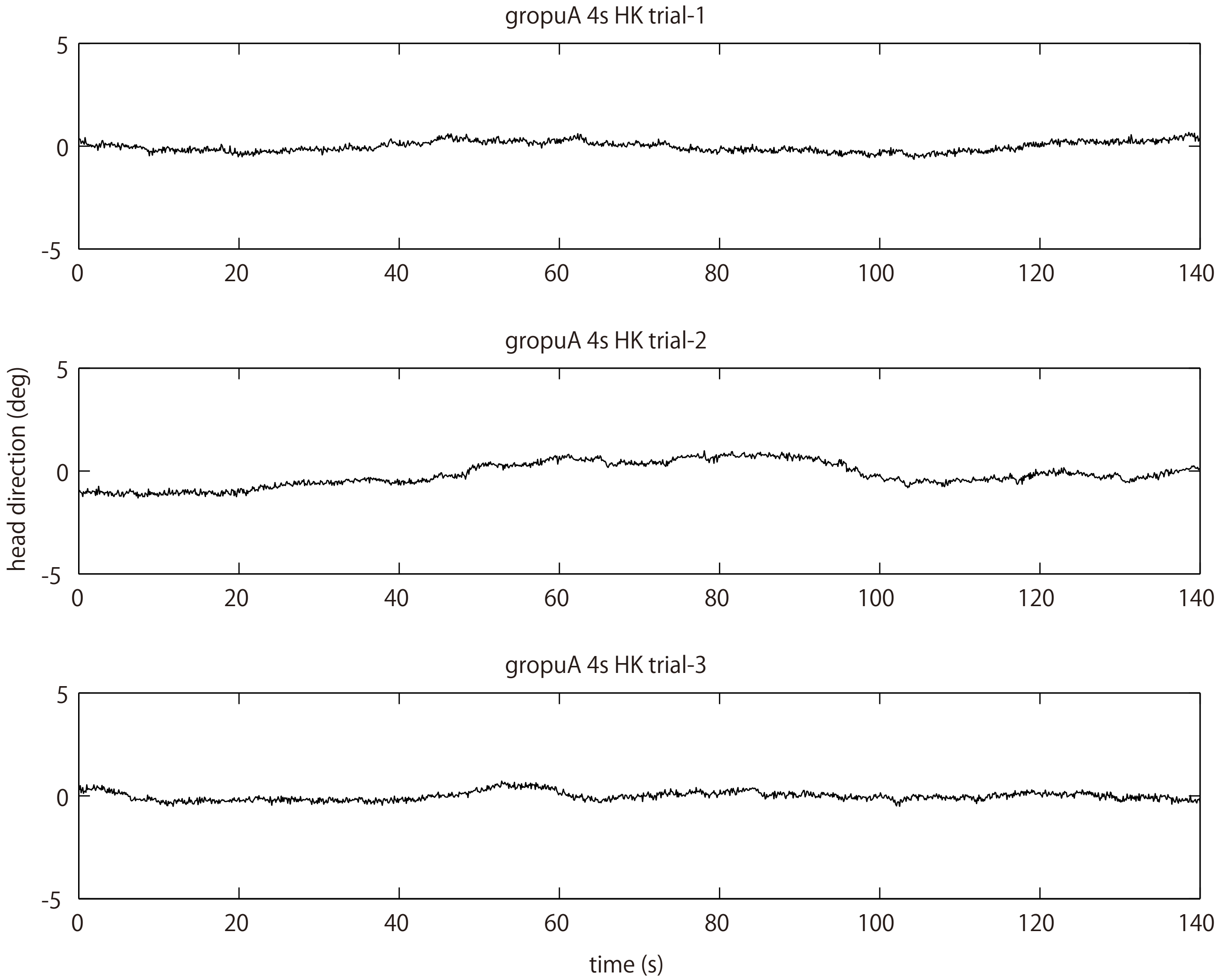
gropuA 4s HK trial-1
gropuA 4s HK trial-2
gropuA 4s HK trial-3
head direction (deg)
time (s)
5
0
-5
0
20
40
60
80
100
120
140

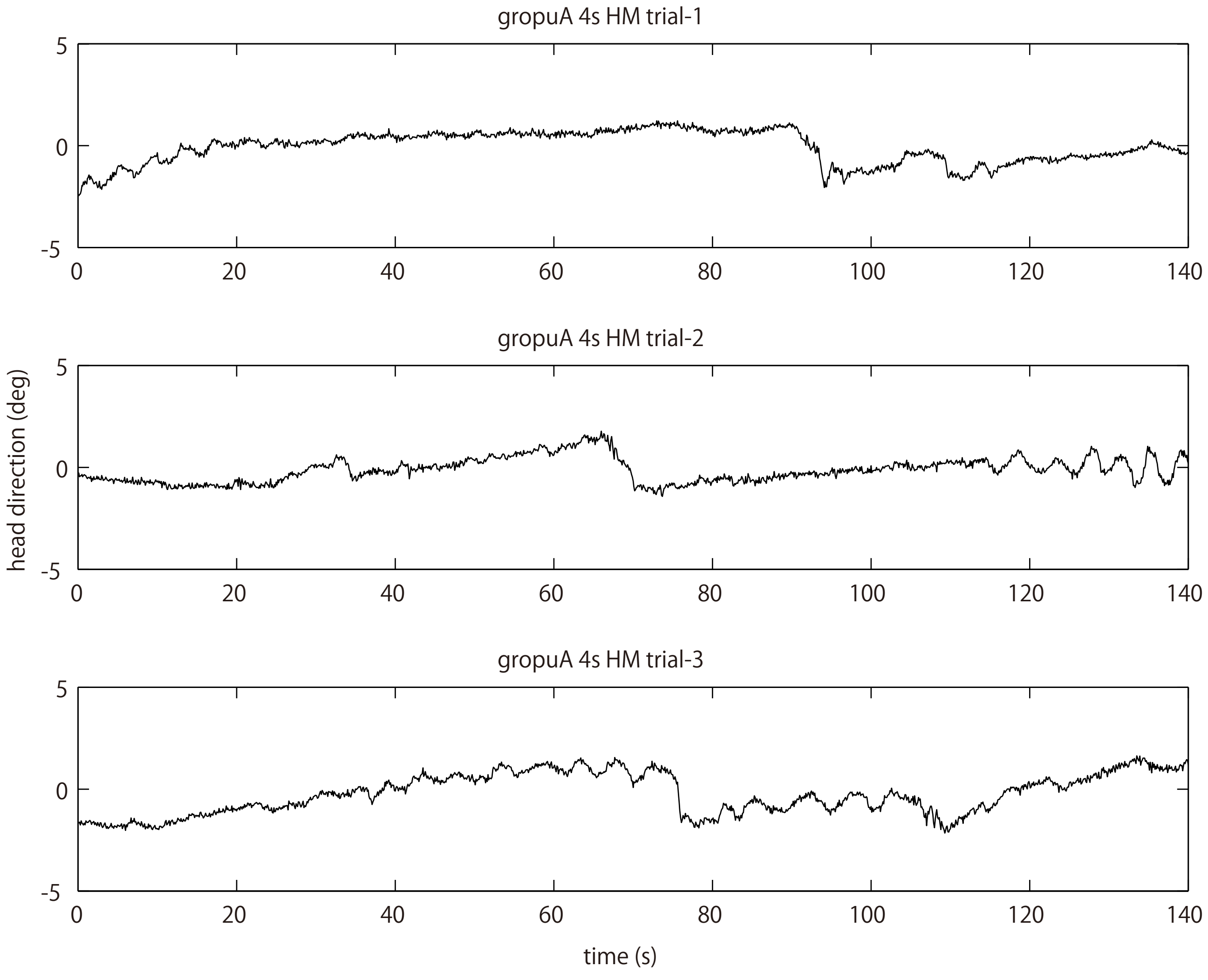
gropuA 4s HM trial-1
gropuA 4s HM trial-2
gropuA 4s HM trial-3
head direction (deg)
time (s)
5
0
-5
0
20
40
60
80
100
120
140

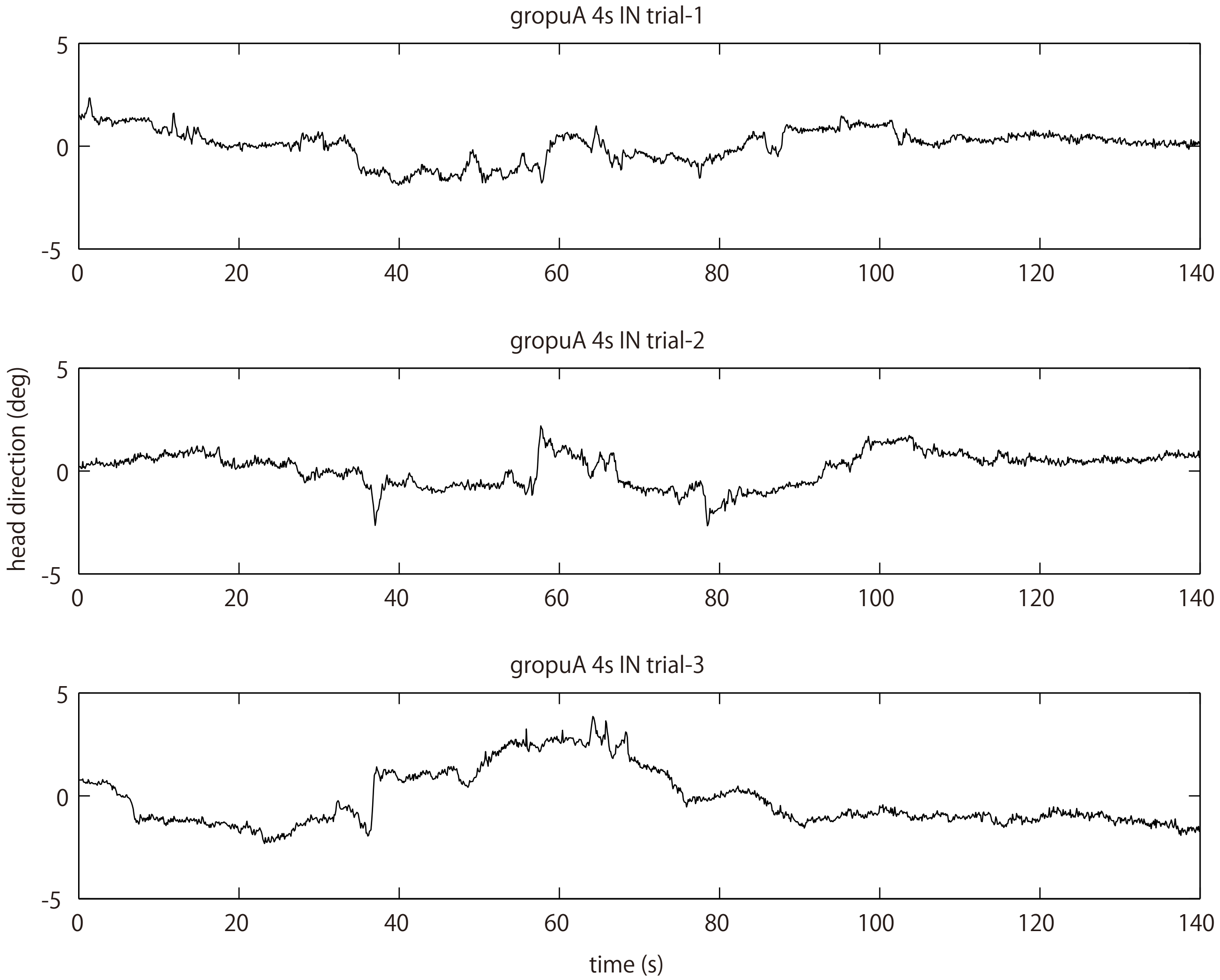
gropuA 4s IN trial-1
gropuA 4s IN trial-2
gropuA 4s IN trial-3
head direction (deg)
time (s)
5
0
-5
0
20
40
60
80
100
120
140

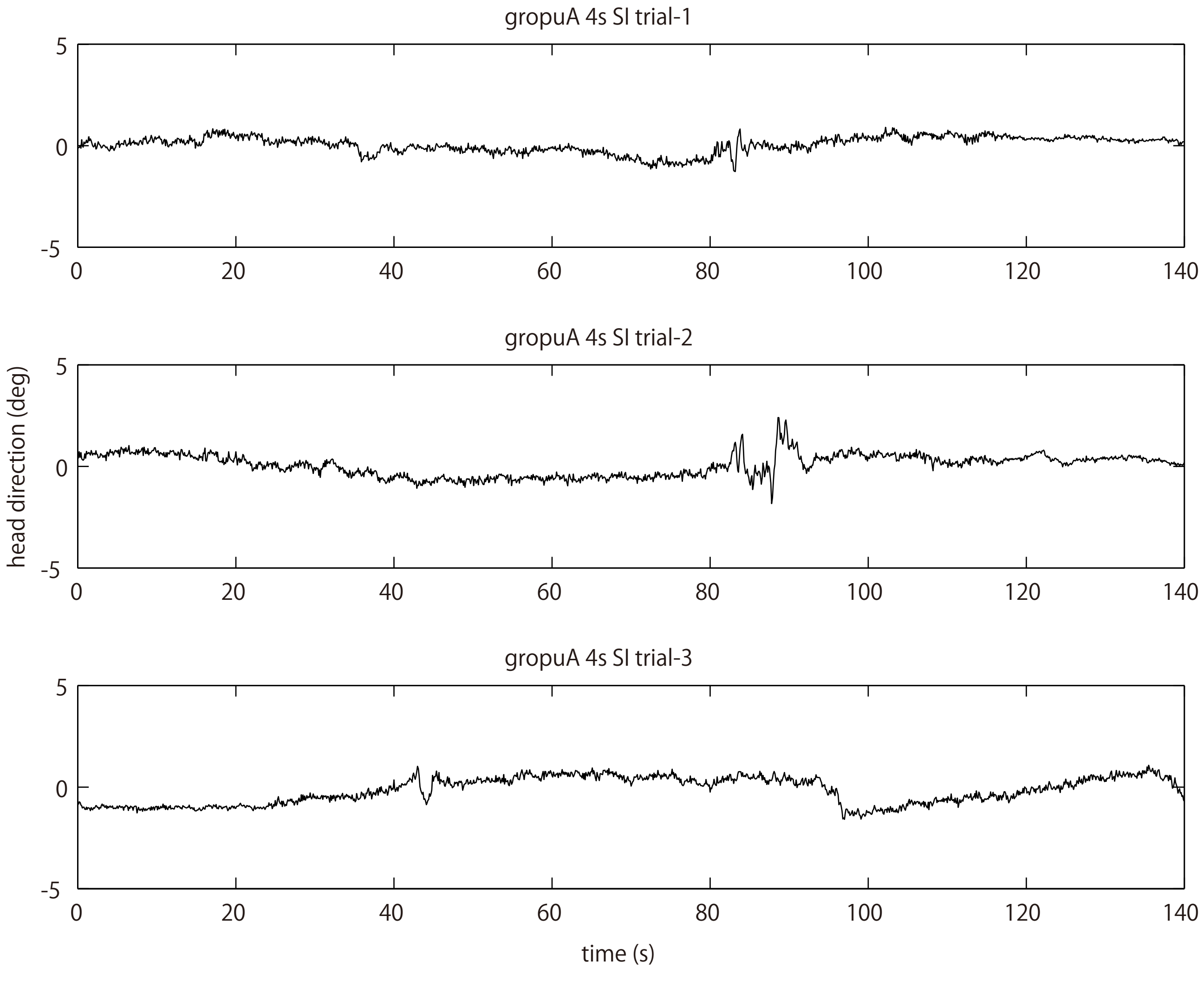
gropuA 4s SI trial-1
gropuA 4s SI trial-2
gropuA 4s SI trial-3
head direction (deg)
time (s)
5
0
-5
0
20
40
60
80
100
120
140

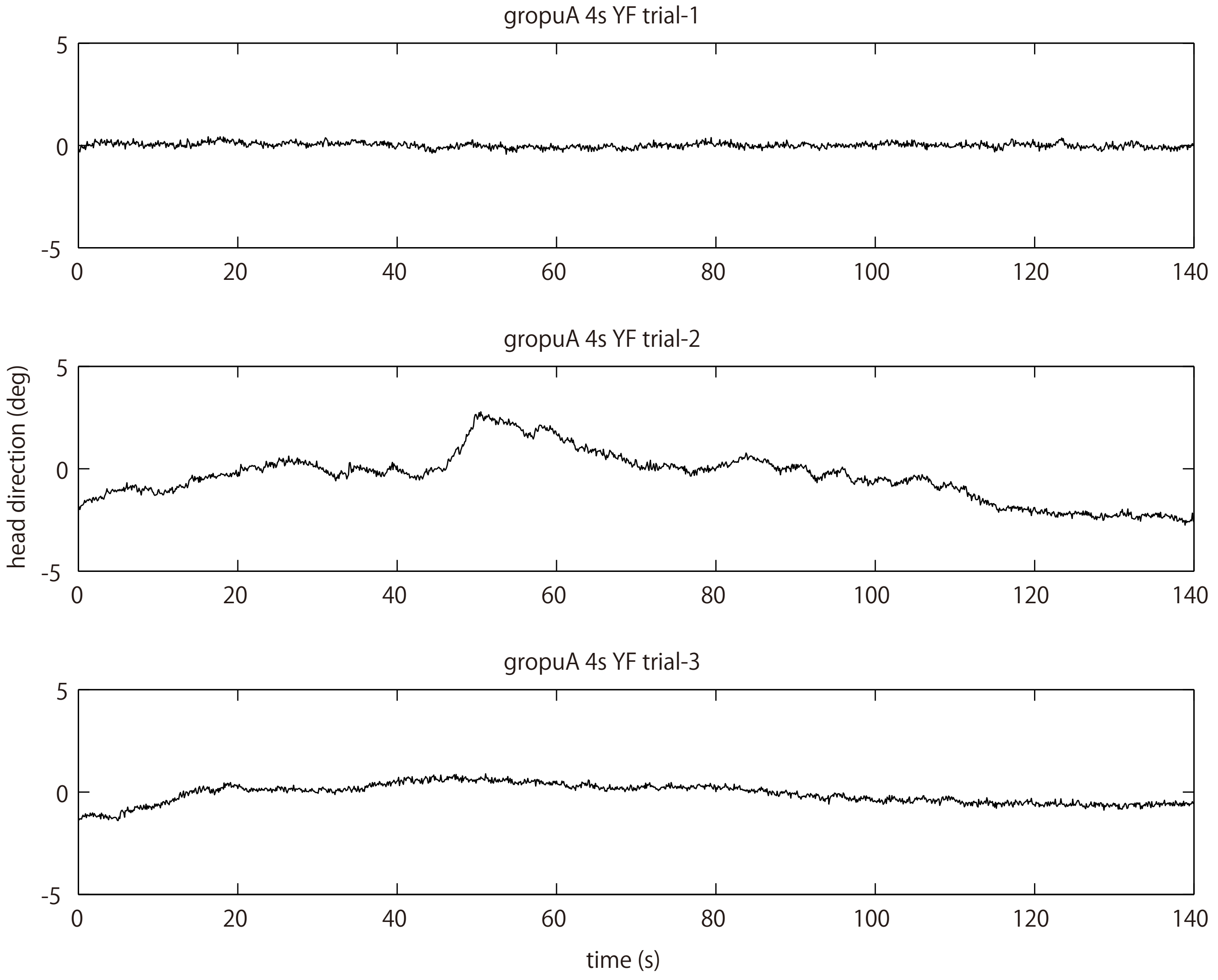
gropuA 4s YF trial-1
gropuA 4s YF trial-2
gropuA 4s YF trial-3
head direction (deg)
time (s)
5
0
-5
0
20
40
60
80
100
120
140

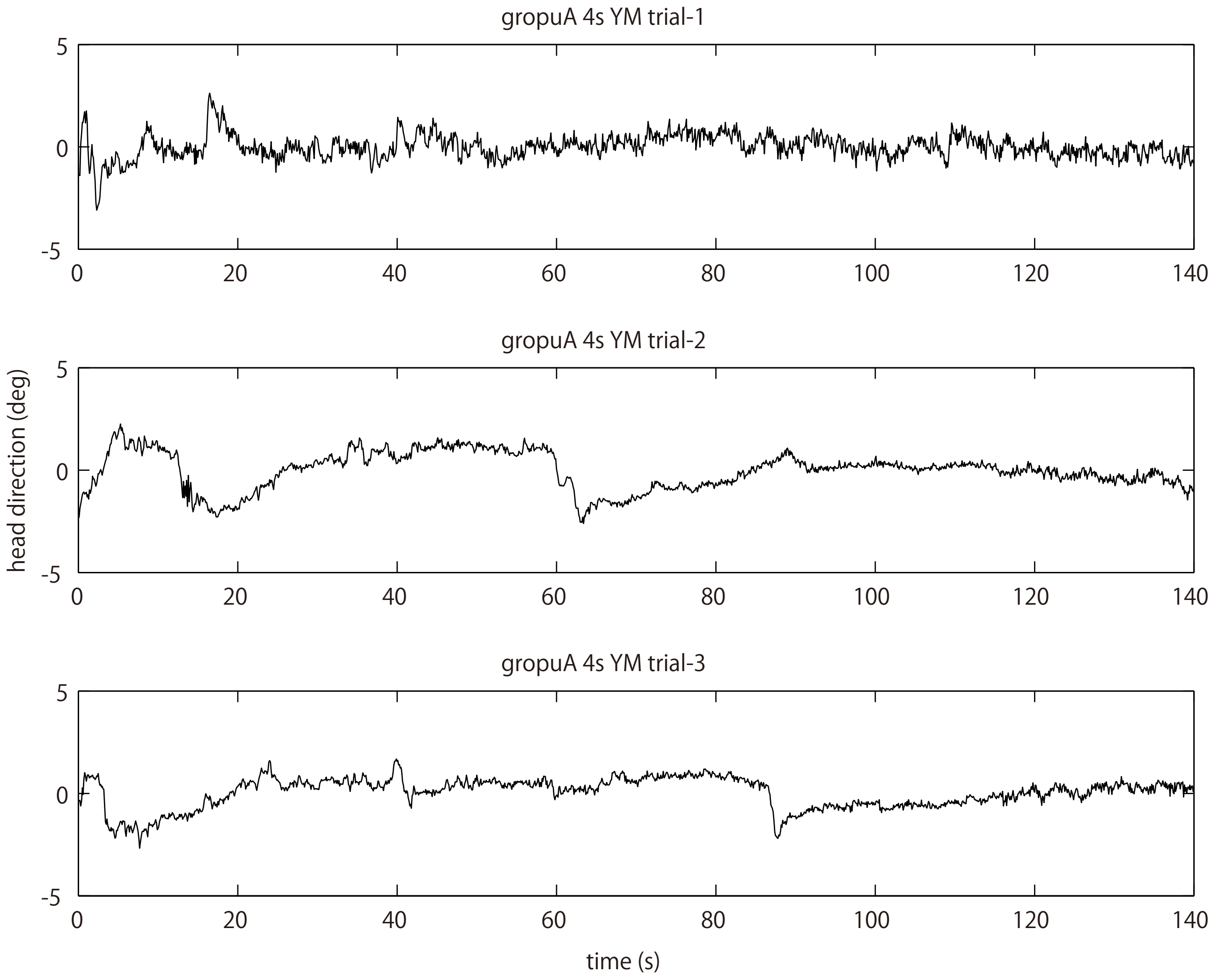
gropuA 4s YM trial-1
gropuA 4s YM trial-2
gropuA 4s YM trial-3
head direction (deg)
time (s)
5
0
-5
0
20
40
60
80
100
120
140

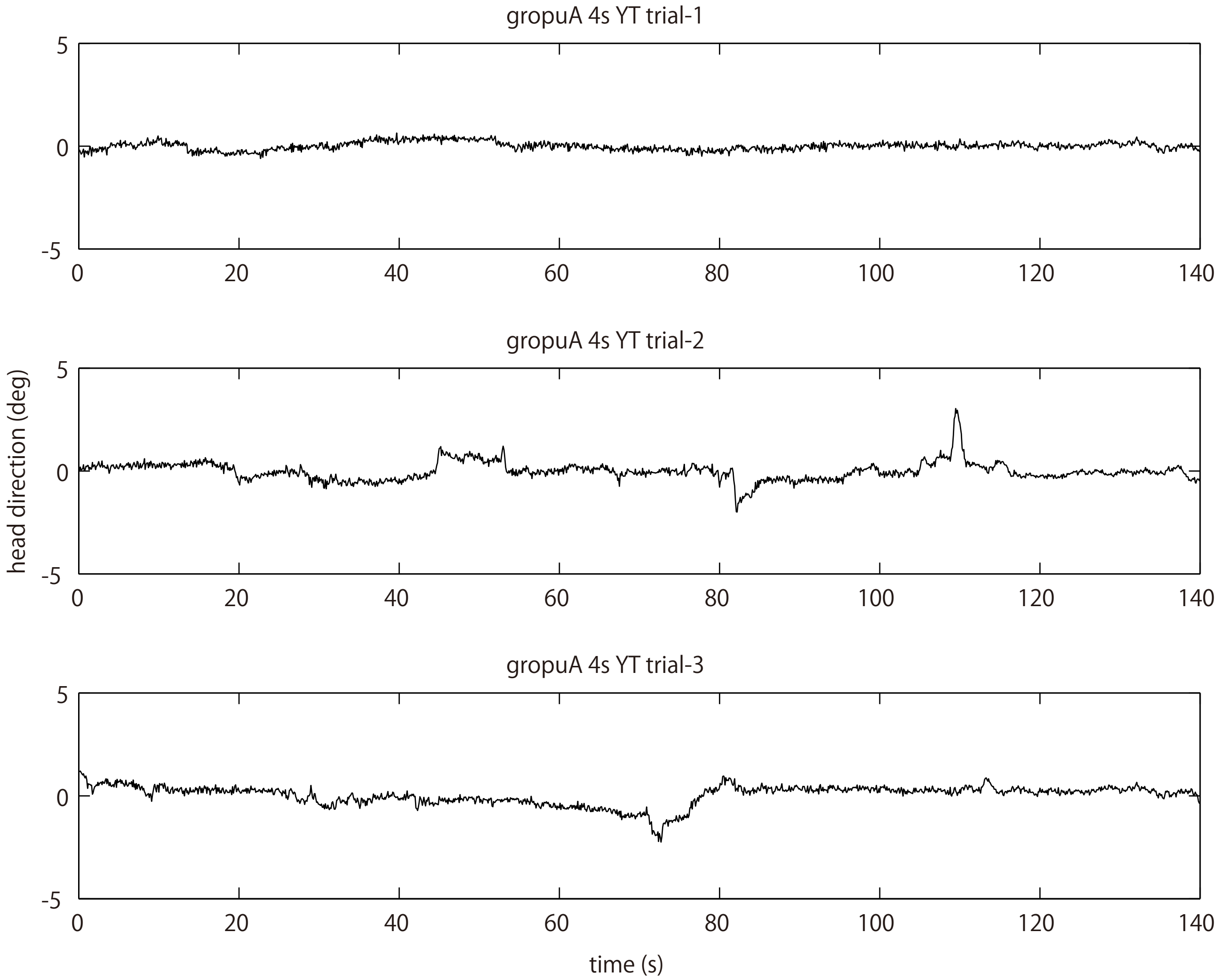

gropuA 4s YT trial-1
gropuA 4s YT trial-2
gropuA 4s YT trial-3
head direction (deg)
time (s)
5
0
-5
0
20
40
60
80
100
120
140

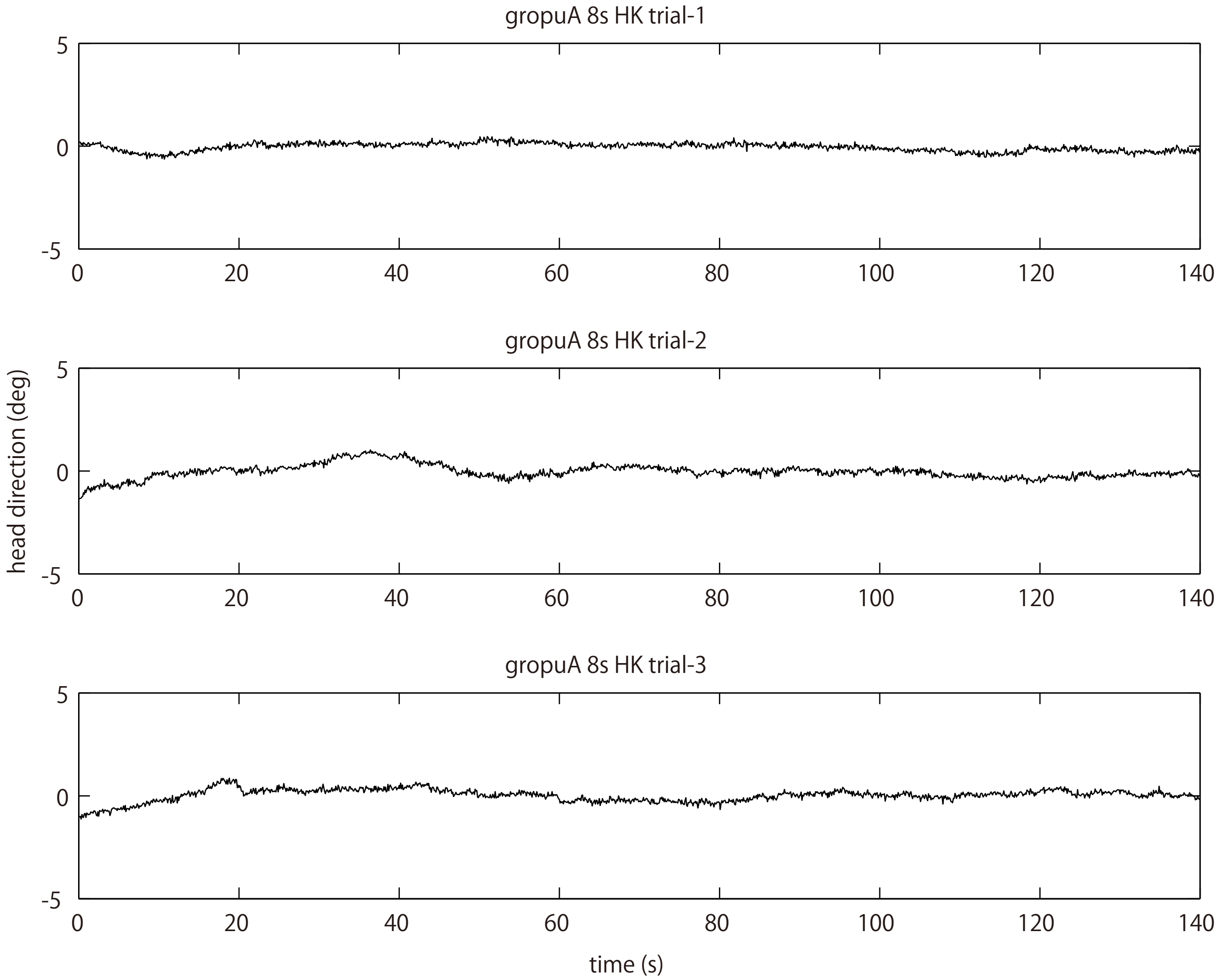
gropuA 8s HK trial-1
gropuA 8s HK trial-2
gropuA 8s HK trial-3
head direction (deg)
time (s)
5
0
-5
0
20
40
60
80
100
120
140

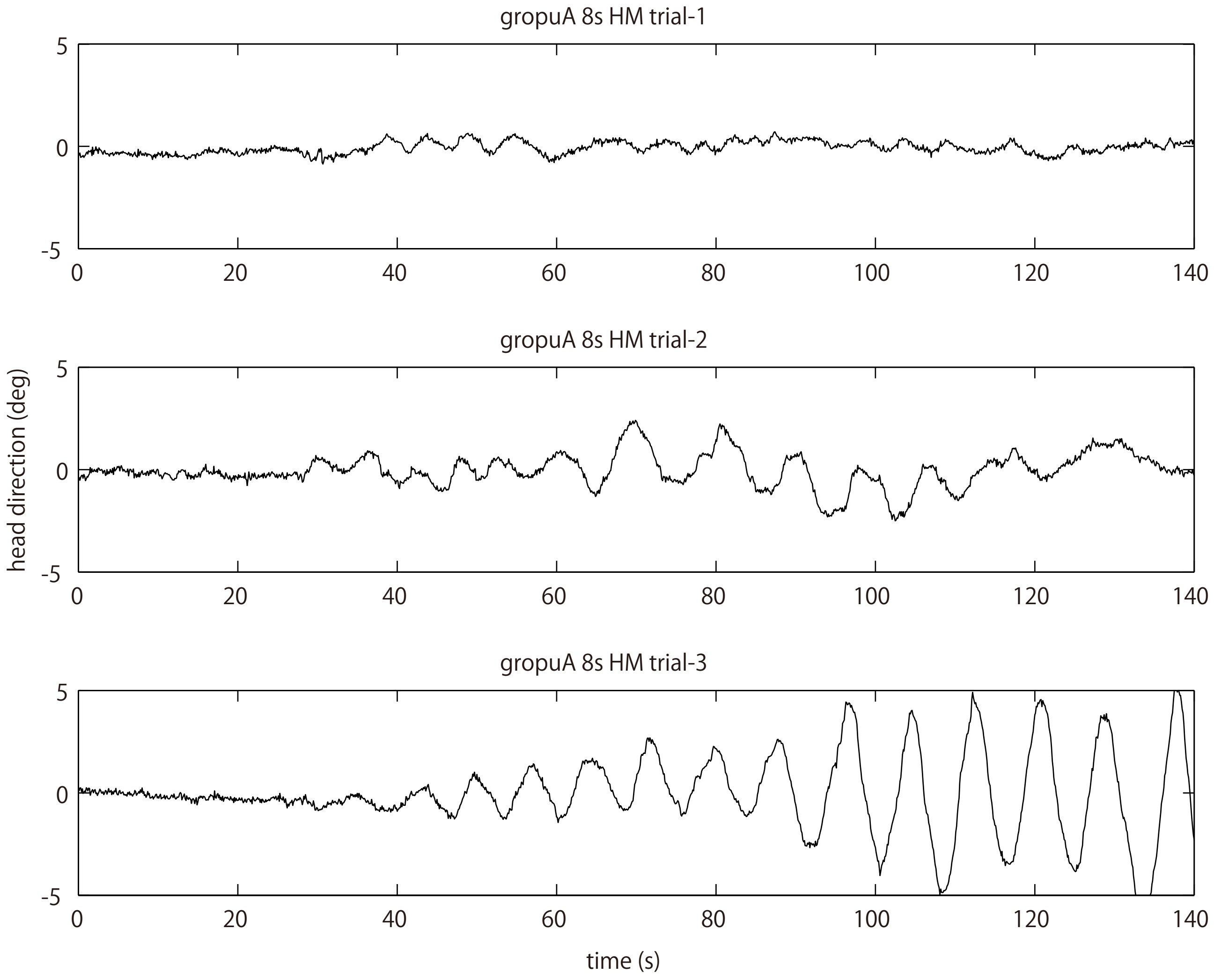
gropuA 8s HM trial-1
gropuA 8s HM trial-2
gropuA 8s HM trial-3
head direction (deg)
time (s)
5
0
-5
0
20
40
60
80
100
120
140

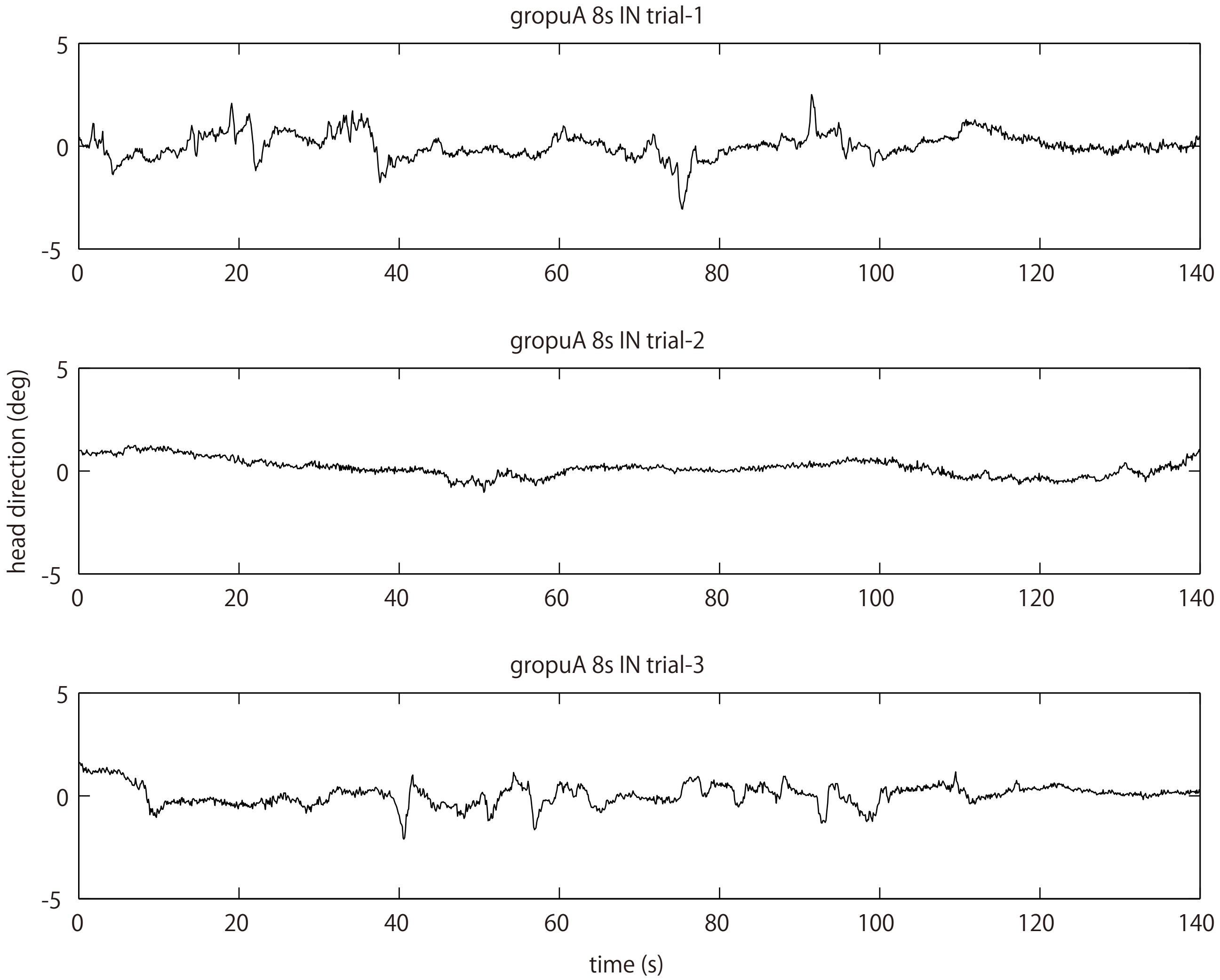
gropuA 8s IN trial-1
gropuA 8s IN trial-2
gropuA 8s IN trial-3
head direction (deg)
time (s)

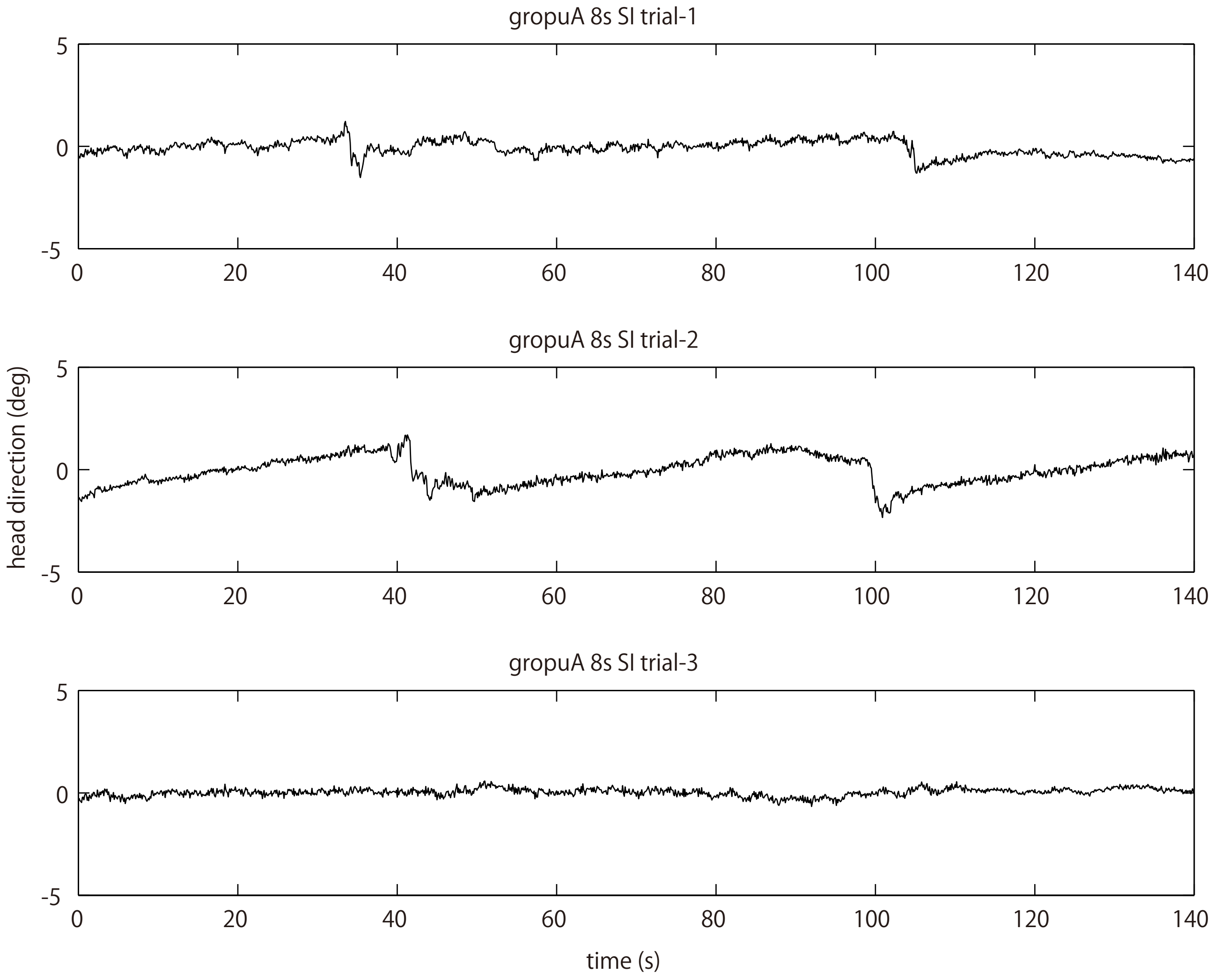
gropuA 8s SI trial-1
gropuA 8s SI trial-2
gropuA 8s SI trial-3
head direction (deg)
time (s)

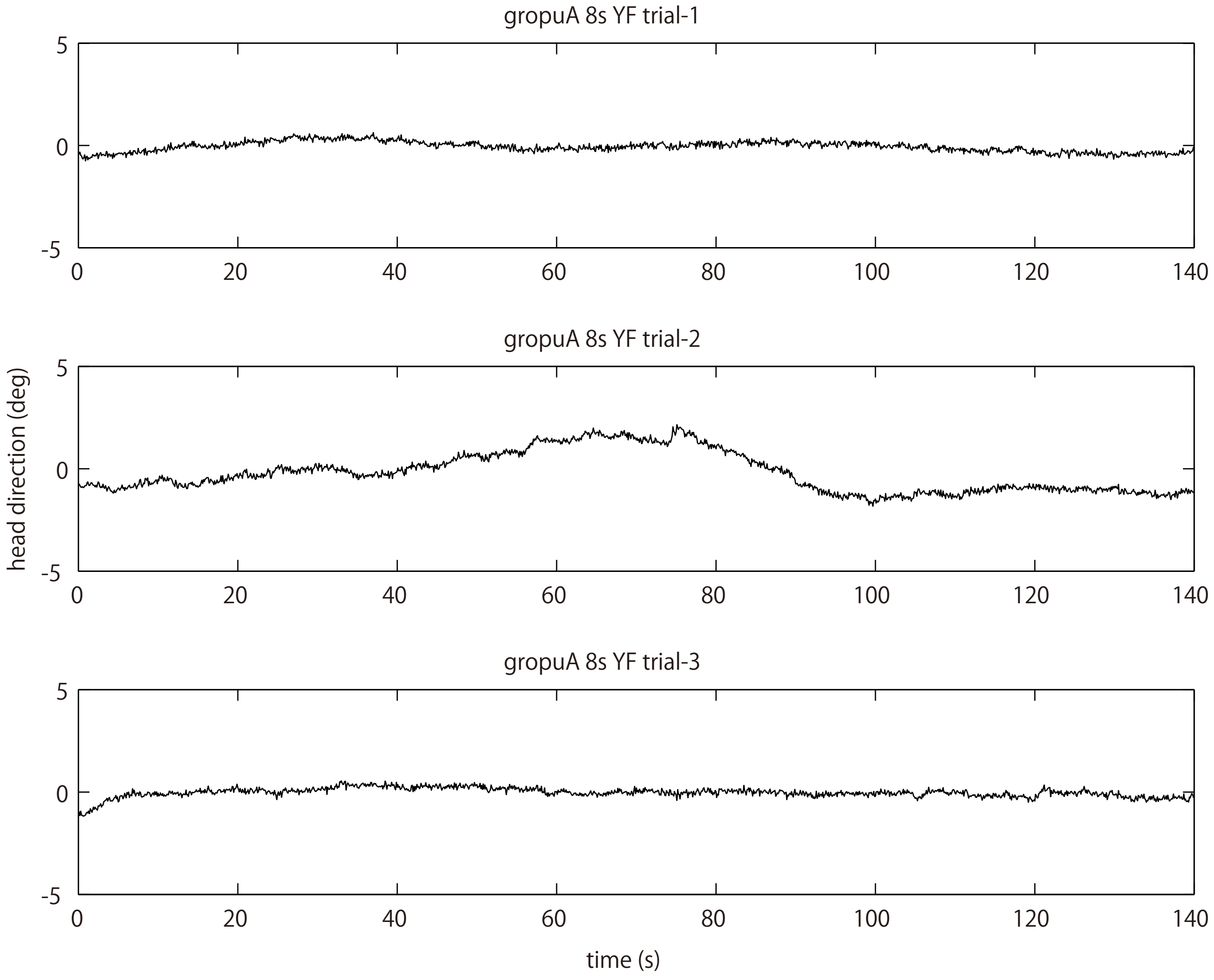
gropuA 8s YF trial-1
gropuA 8s YF trial-2
gropuA 8s YF trial-3
head direction (deg)
time (s)
5
0
-5
0
20
40
60
80
100
120
140

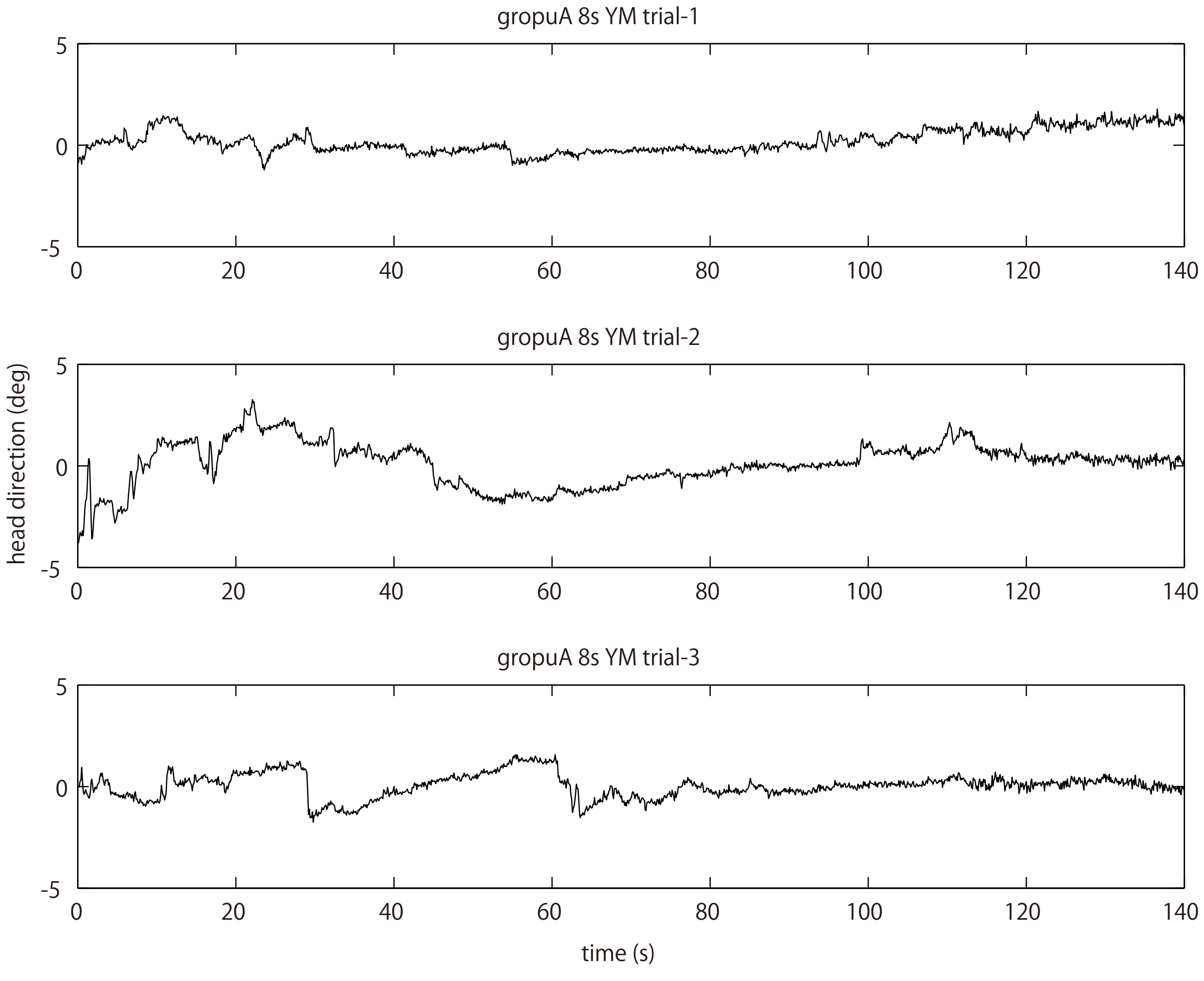
gropuA 8s YM trial-1
gropuA 8s YM trial-2
gropuA 8s YM trial-3
head direction (deg)
time (s)
5
0
-5
0
20
40
60
80
100
120
140

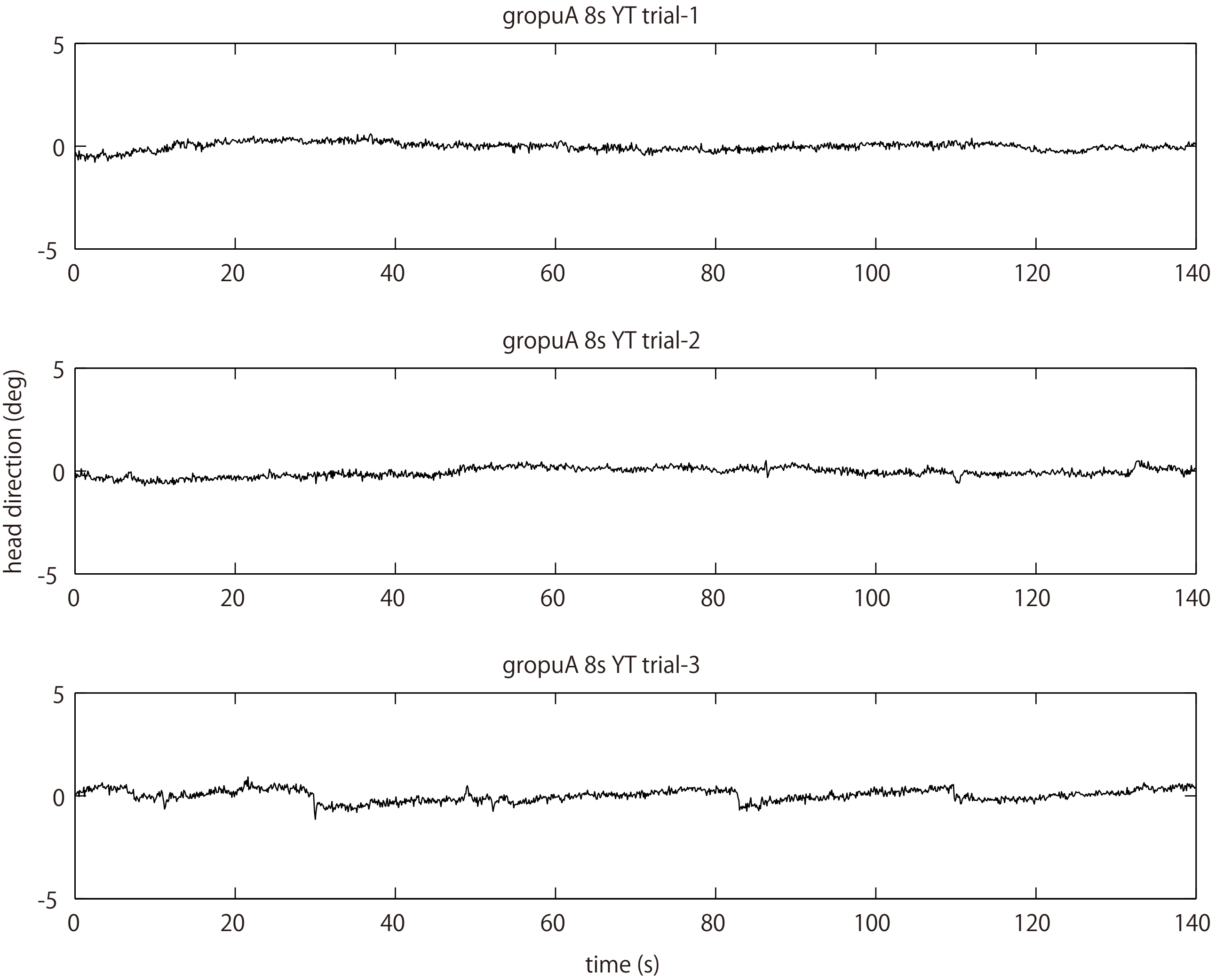
gropuA 8s YT trial-1
gropuA 8s YT trial-2
gropuA 8s YT trial-3
5
0
-5
0
20
40
60
80
100
120
140
head direction (deg)
time (s)

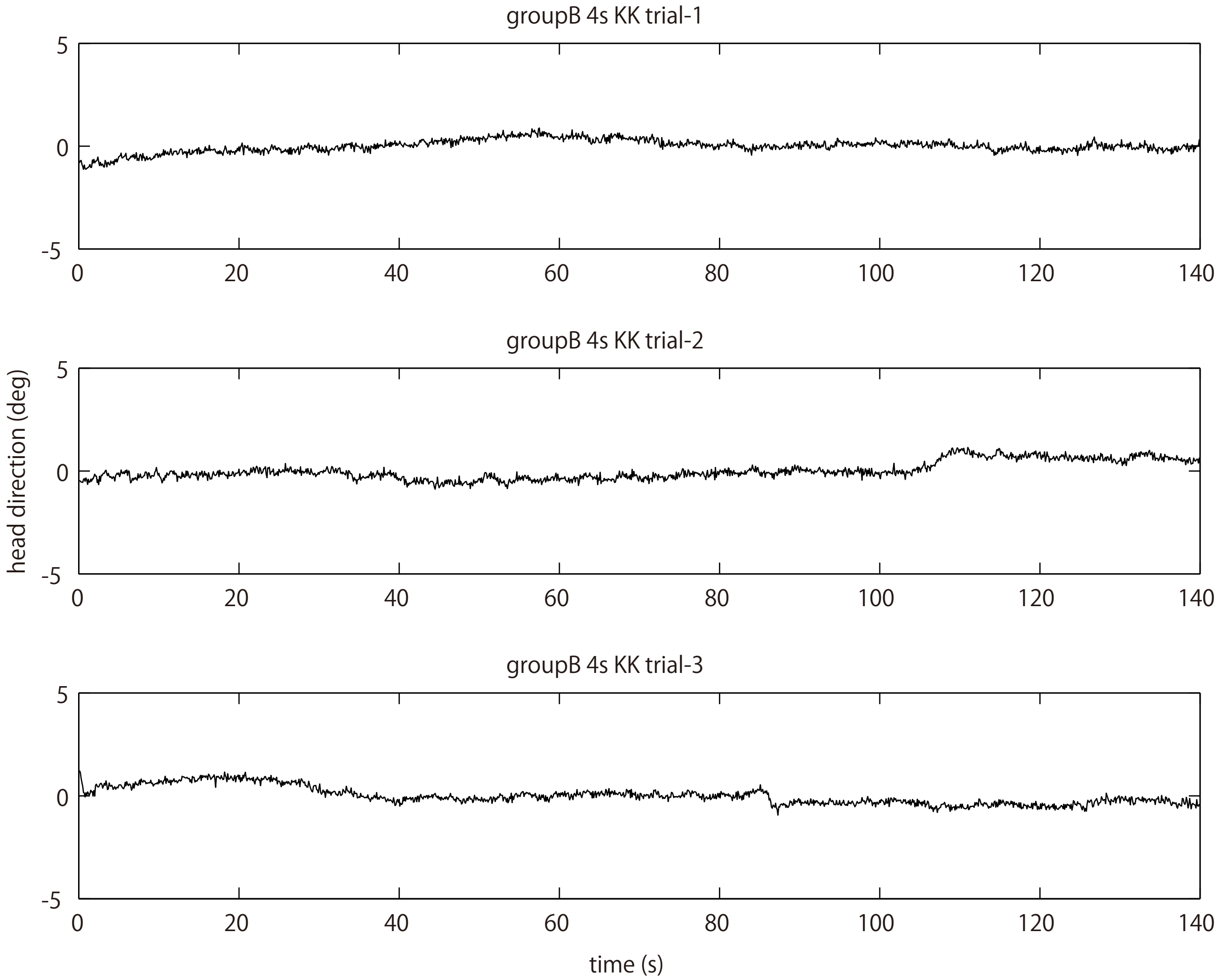
groupB 4s KK trial-1
groupB 4s KK trial-2
groupB 4s KK trial-3
head direction (deg)
time (s)

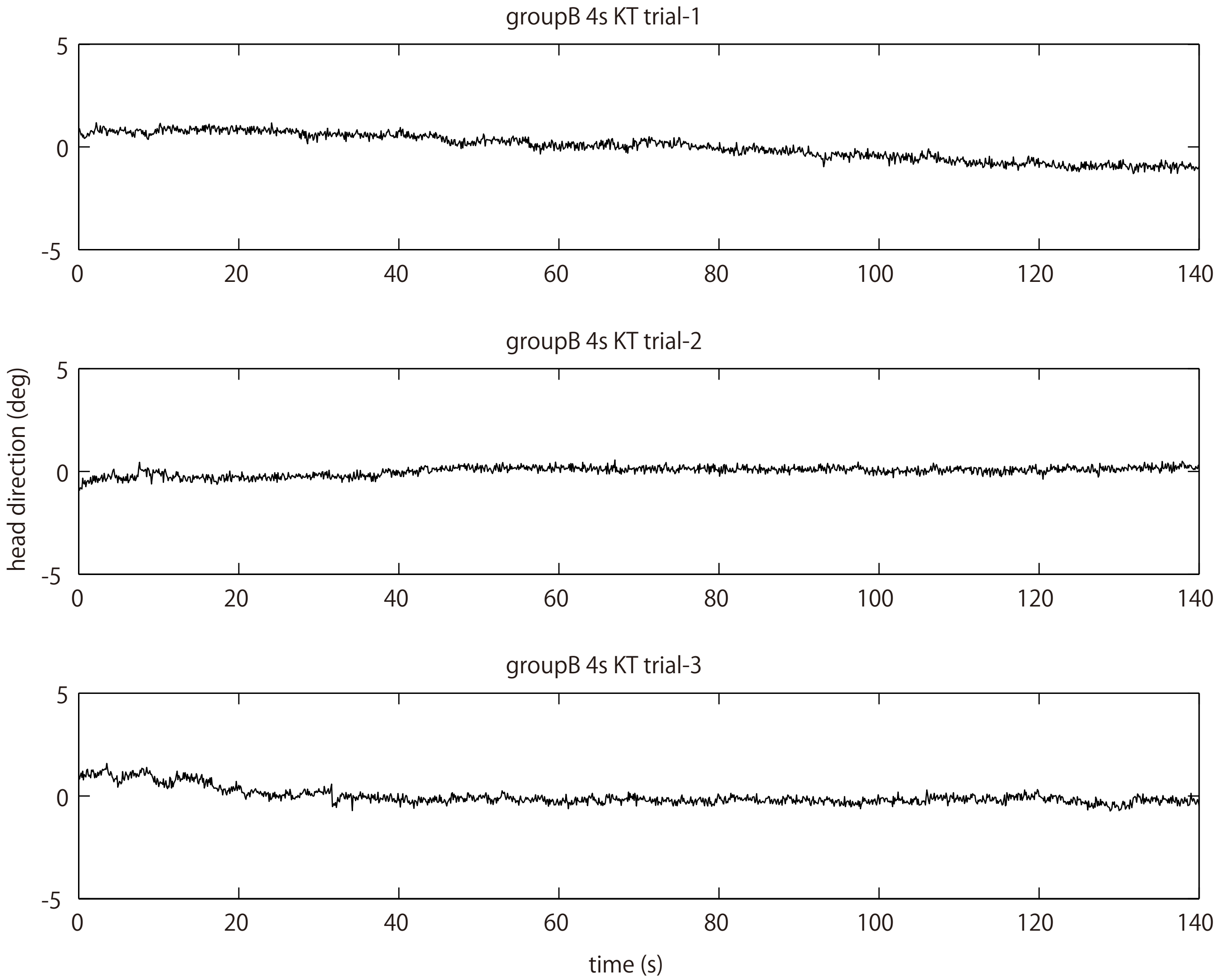
groupB 4s KT trial-1
groupB 4s KT trial-2
groupB 4s KT trial-3
head direction (deg)
time (s)

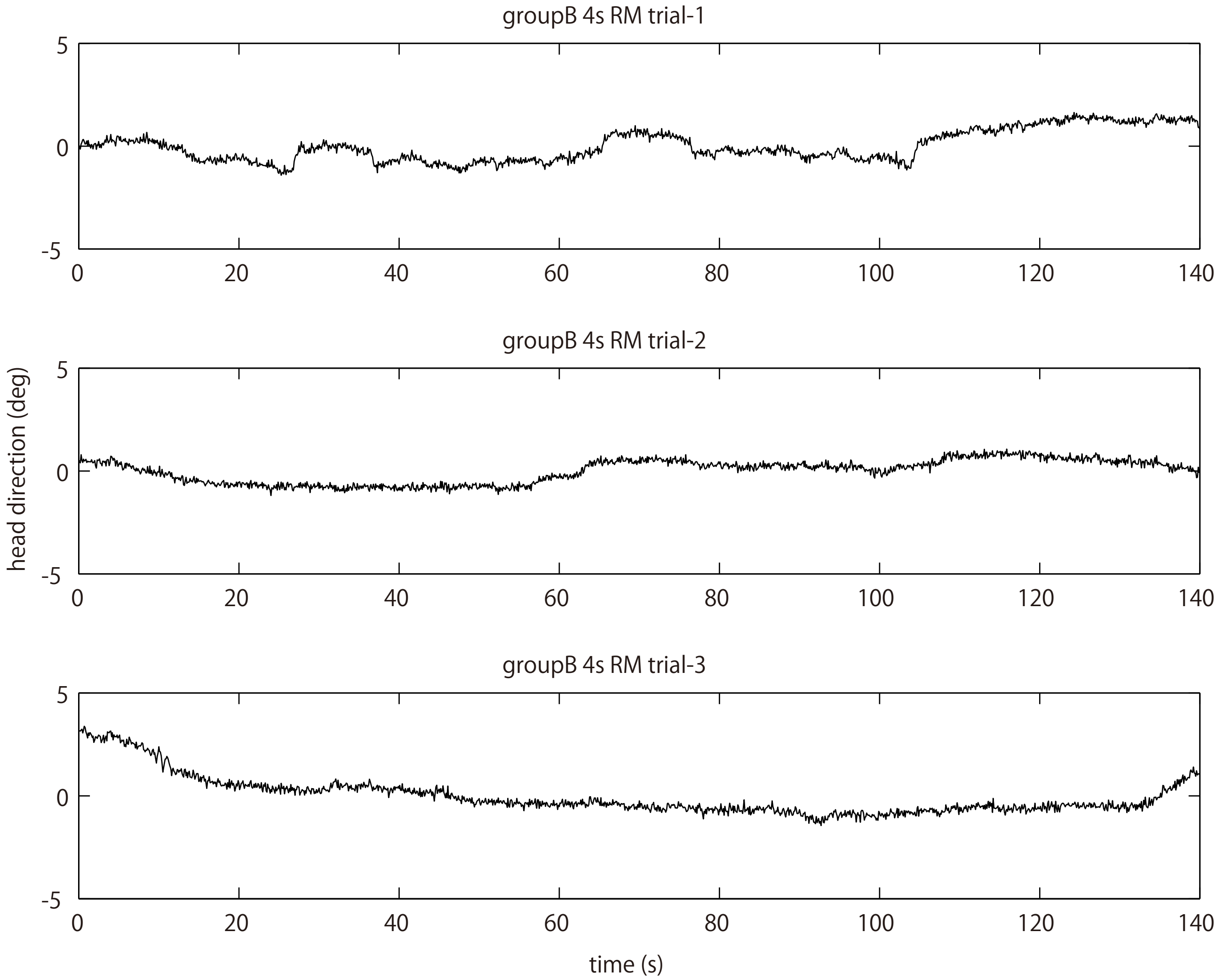
groupB 4s RM trial-1
groupB 4s RM trial-2
groupB 4s RM trial-3
head direction (deg)
time (s)
5
0
-5
0
20
40
60
80
100
120
140

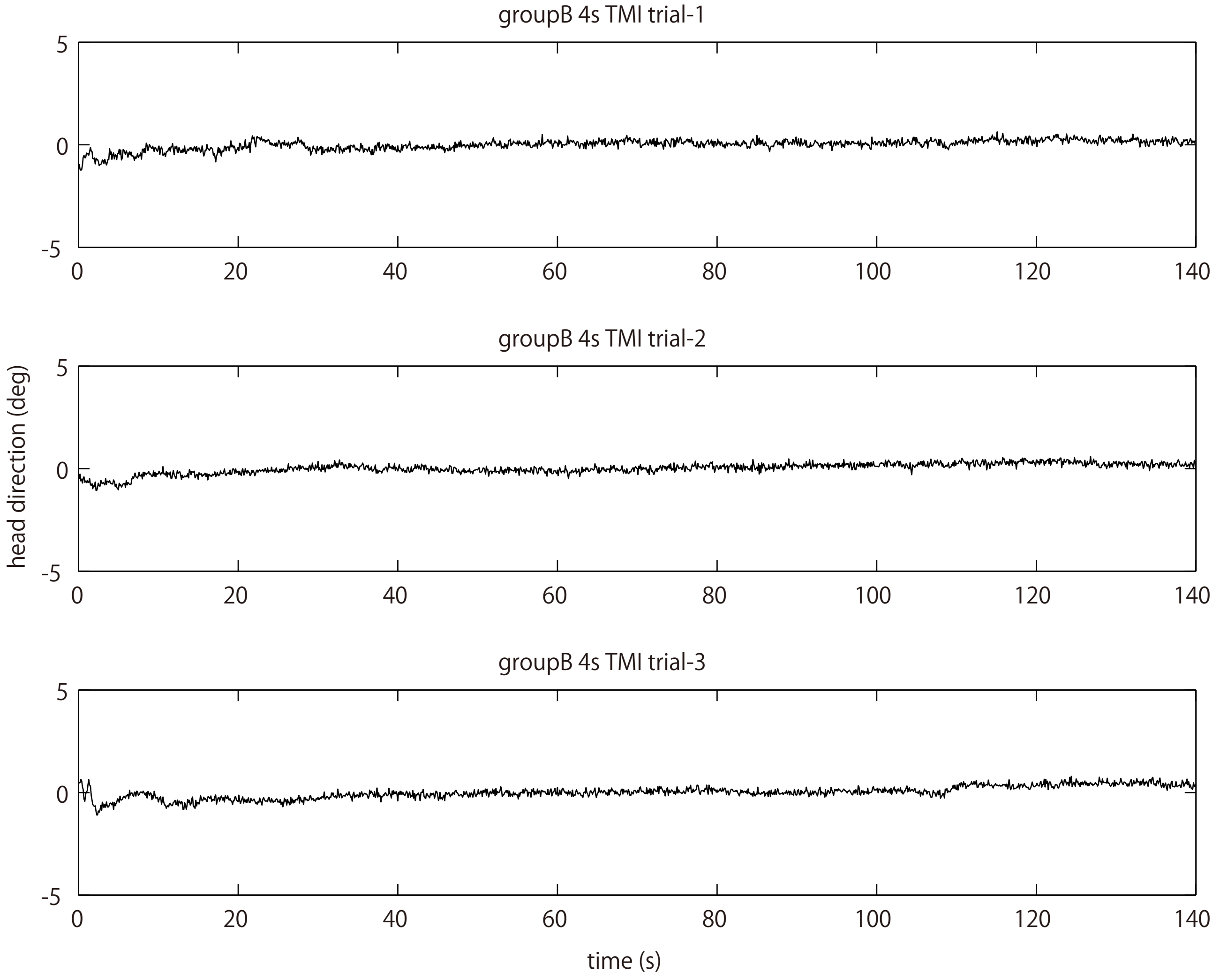
groupB 4s TMI trial-1
groupB 4s TMI trial-2
groupB 4s TMI trial-3
head direction (deg)
time (s)
5
0
-5
0
20
40
60
80
100
120
140

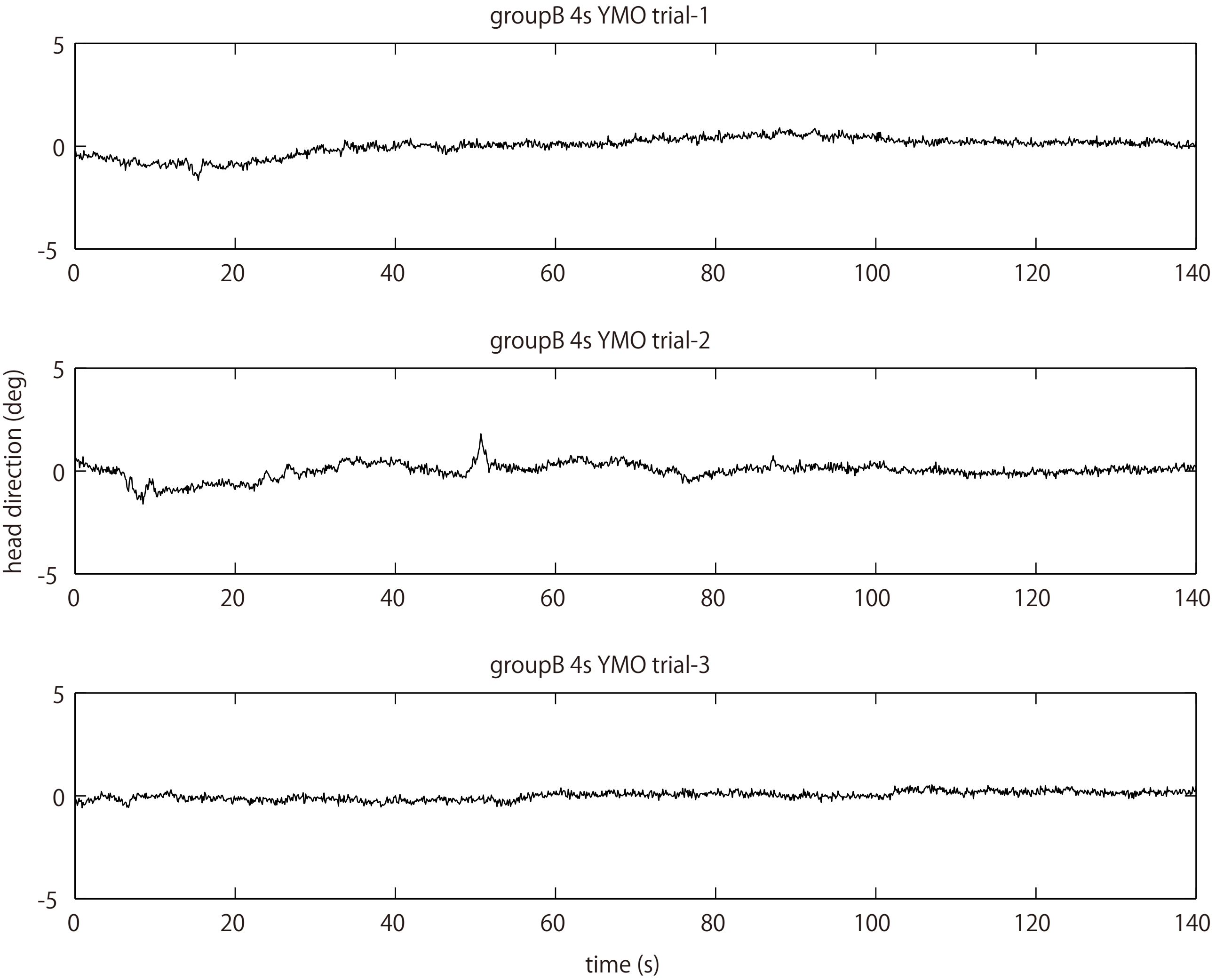
groupB 4s YMO trial-1
groupB 4s YMO trial-2
groupB 4s YMO trial-3
head direction (deg)
time (s)
5
0
-5
0
20
40
60
80
100
120
140

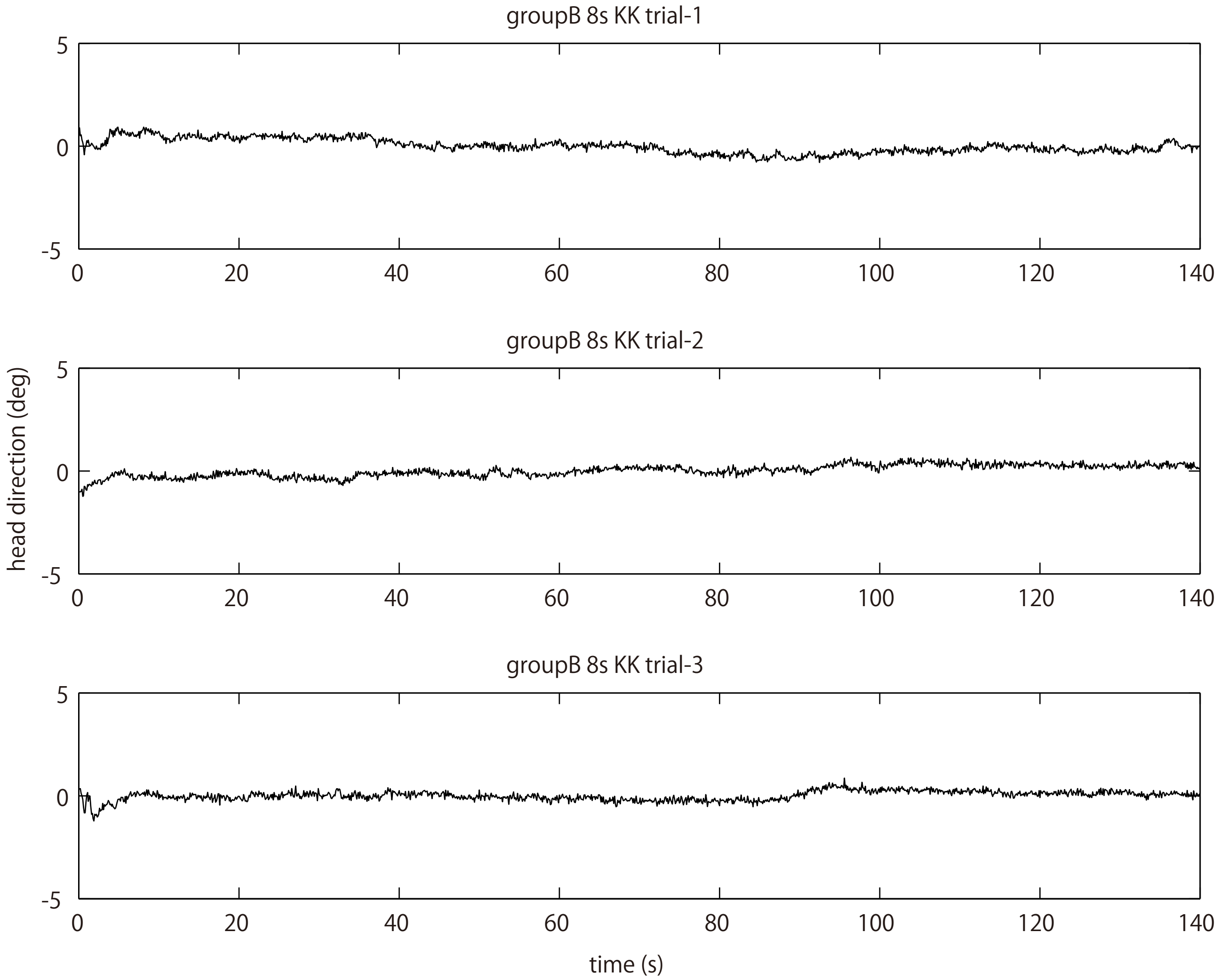
groupB 8s KK trial-1
groupB 8s KK trial-2
groupB 8s KK trial-3
head direction (deg)
time (s)
5
0
-5
0
20
40
60
80
100
120
140

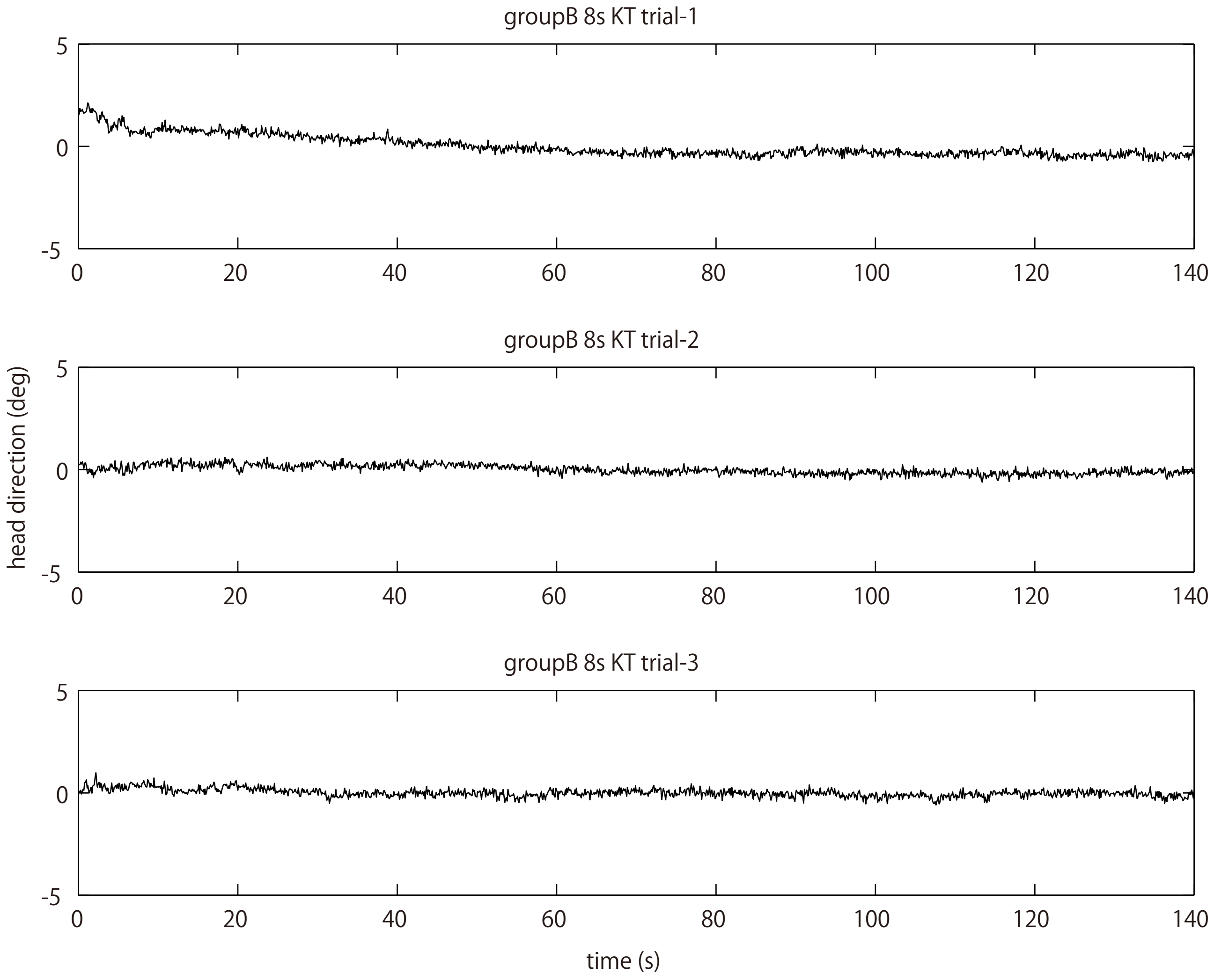
groupB 8s KT trial-1
groupB 8s KT trial-2
groupB 8s KT trial-3
head direction (deg)
time (s)
5
0
-5
0
20
40
60
80
100
120
140

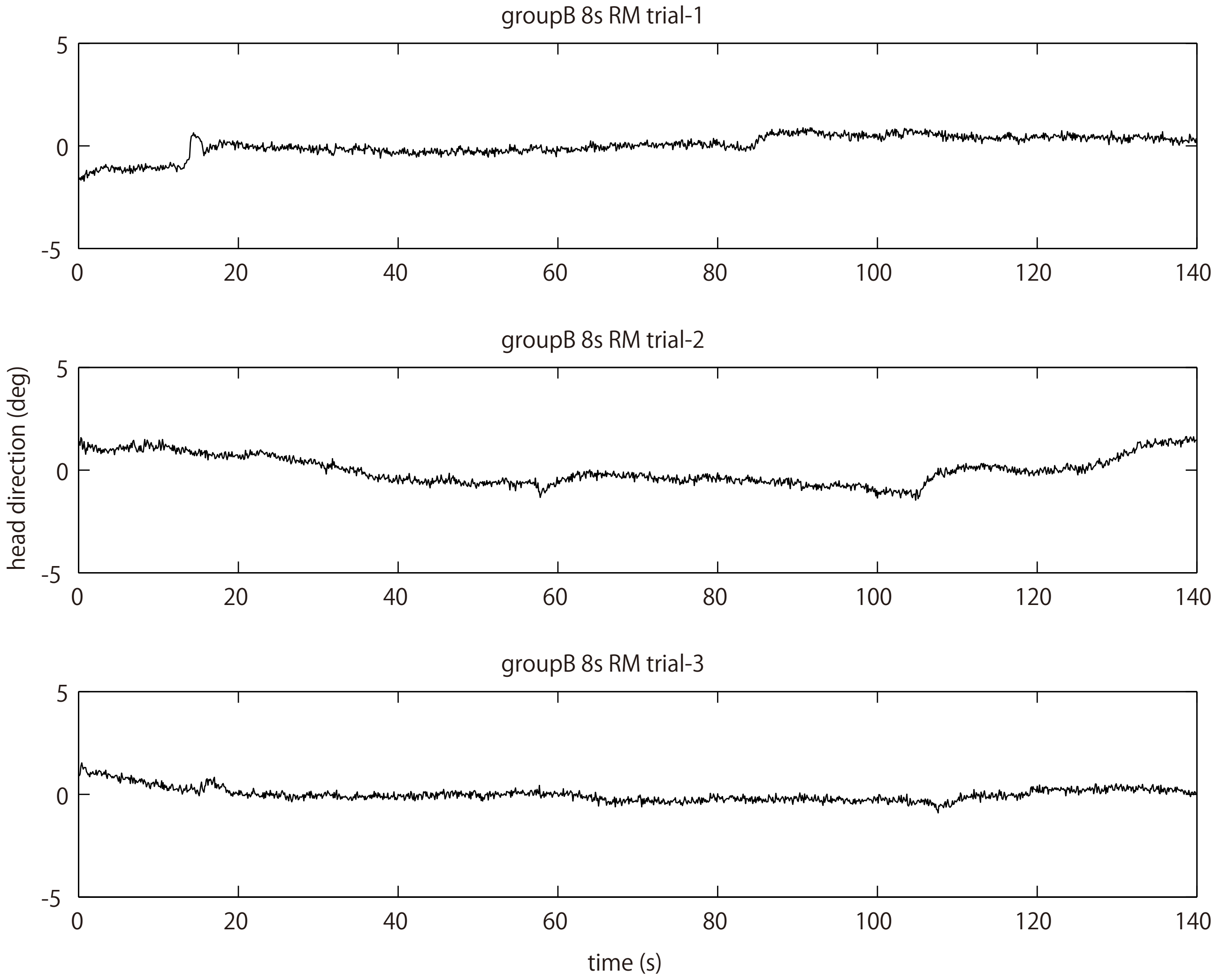
groupB 8s RM trial-1
groupB 8s RM trial-2
groupB 8s RM trial-3
head direction (deg)
time (s)
5
0
-5
0
20
40
60
80
100
120
140

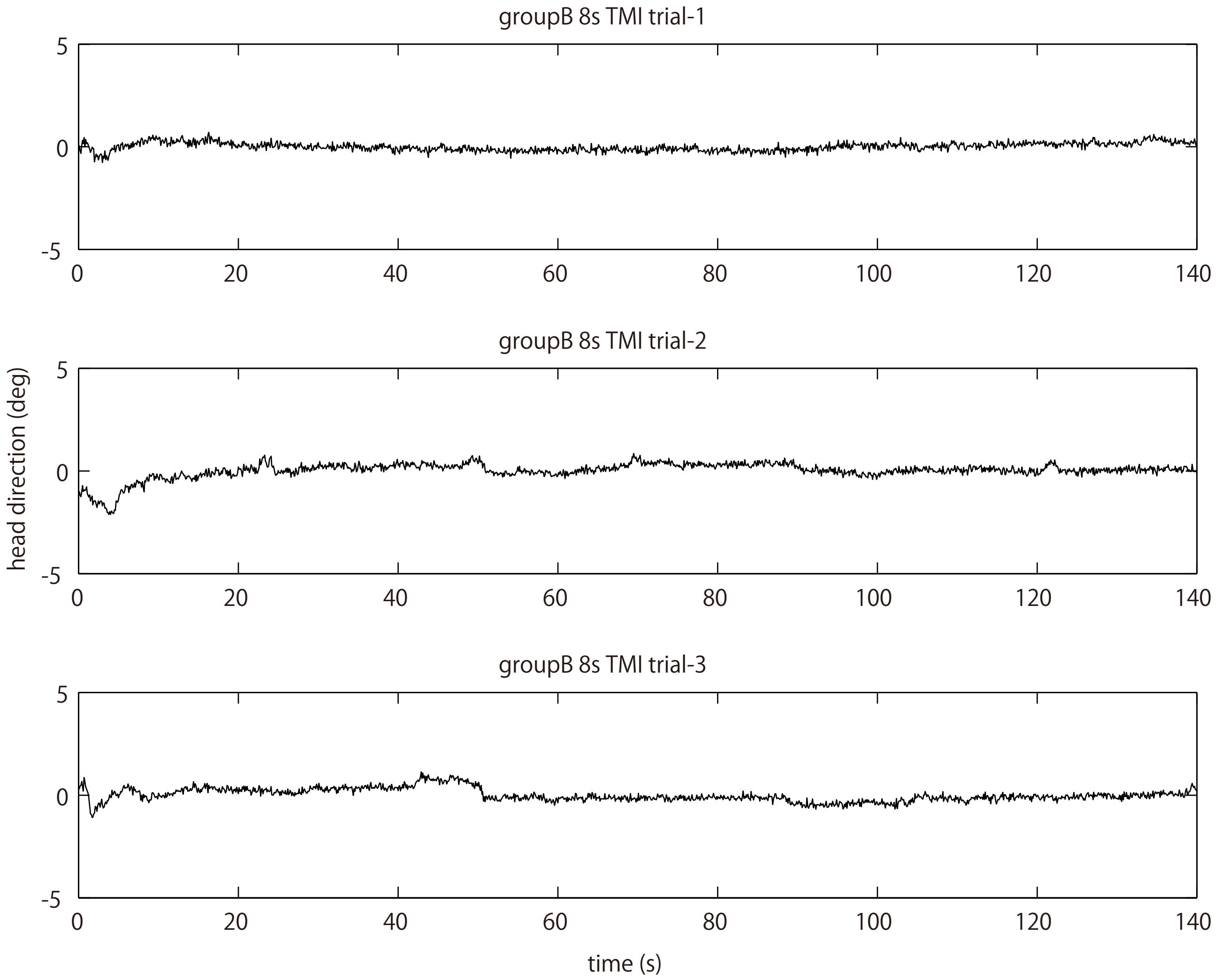
groupB 8s TMI trial-1
groupB 8s TMI trial-2
groupB 8s TMI trial-3
head direction (deg)
time (s)
5
0
-5
0
20
40
60
80
100
120
140

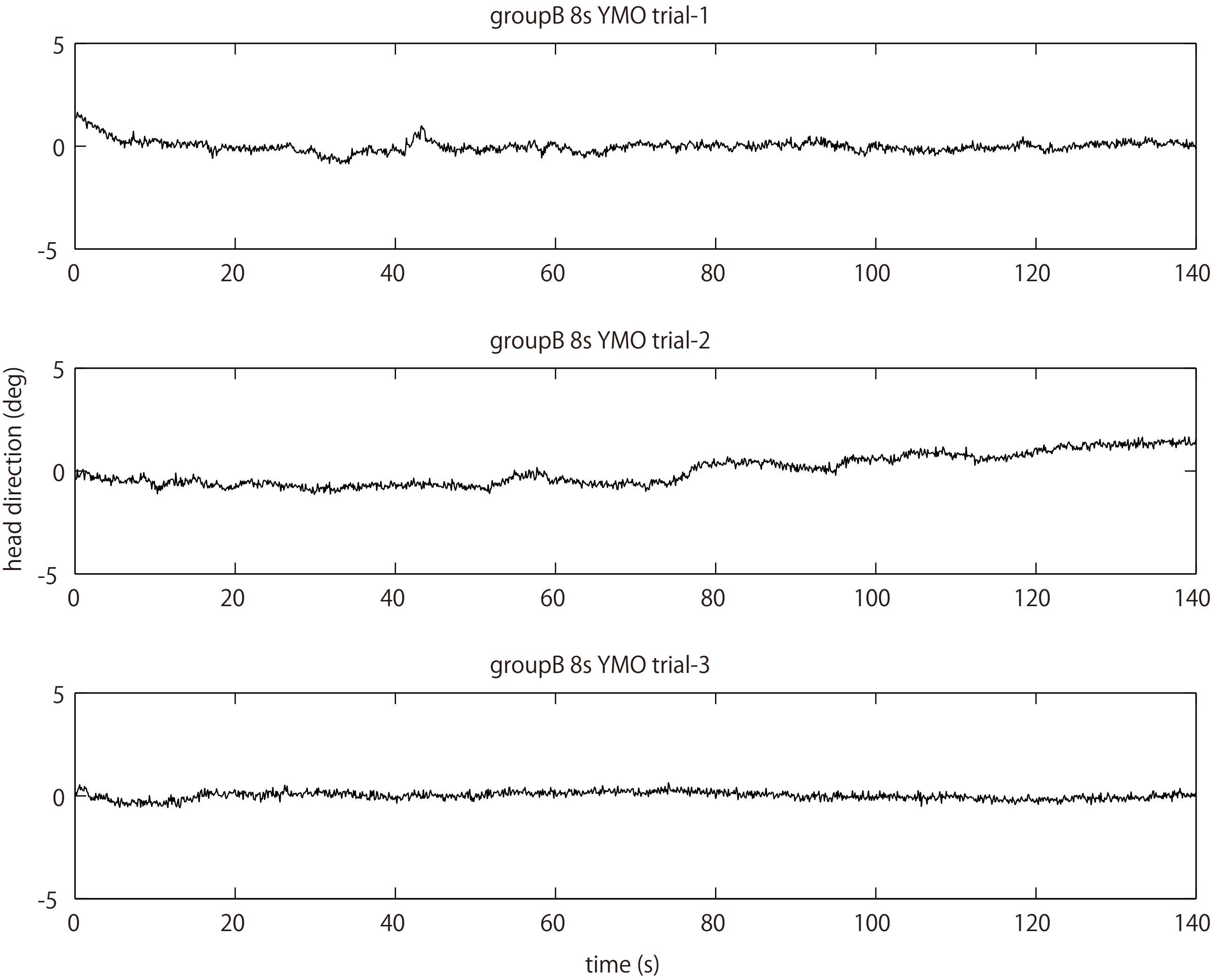

groupB 8s YMO trial-1
groupB 8s YMO trial-2
groupB 8s YMO trial-3
head direction (deg)
time (s)
5
0
-5
0
20
40
60
80
100
120
140
